# In vitro effect of fluoride-free mouthwashes on *Streptococcus mutans* biofilm

**DOI:** 10.1007/s00784-025-06462-7

**Published:** 2025-08-14

**Authors:** Astrid C. Valdivia-Tapia, Frank Lippert, Peter F. Castelluccio, Jaime A. Cury, Antonio P. Ricomini-Filho, Richard L. Gregory

**Affiliations:** 1 Department of Biomedical and Applied Sciences, Indiana University School of Dentistry, Indianapolis, IN 46202 USA; 2 Department of Biostatistics, Indiana University School of Medicine, Indianapolis, IN USA; 3 Piracicaba Dental School, University of Campinas, Piracicaba, SP Brazil; 4 Pathology and Laboratory Medicine, School of Medicine, Indianapolis, IN USA

**Keywords:** Mouthwash, *S. mutans*, Dental caries, Biofilm, Antibacterial

## Abstract

**Objective:**

To evaluate the efficacy of commercially available, fluoride-free mouthwashes sold in Indianapolis, IN, on *Streptococcus mutans* biofilm.

**Materials and methods:**

Eighty-one different mouthwashes were purchased. A 16-h culture of *S. mutans* UA159 was treated with the mouthwashes in three dilutions (1:3, 1:6, and 1:12), prepared in Tryptic Soy broth supplemented with 1% sucrose. The minimum inhibitory concentrations (MIC), planktonic, and biofilm growth were evaluated using a spectrophotometer. In addition, the growth for minimum bactericidal concentration (MBC) was evaluated using five μL of the dilution and incubated on blood agar. For the analysis of the results, the mouthwashes were separated into six groups according to their active ingredients (cetylpyridinium chloride/CPC, n = 25; essential oils/EO n = 10; whitening/W (hydrogen peroxide/ sodium hexametaphosphate), n = 12; Natural-Derived Actives / NDA, n = 15; zinc chloride/ZC, n = 3; others/O, n = 16). ANOVA followed by the Tukey test was performed (p < 0.05).

**Results:**

Regarding MIC, planktonic, and biofilm growth of *S. mutans*, there was a significant decrease for the W and CPC groups (p < 0.001). The EO and W groups had more inhibition on *S. mutans* biofilm compared to the CPC group (p < 0.05). For ZC, NDA, and O groups, there were different effects within the same group, presenting a large variability. About MBC, W and CPC groups presented the higher inhibition (W > CPC > EO > NDA/ZC/O).

**Conclusion:**

The mouthwashes demonstrated significant effect on S. *mutans* biofilm, especially in the 1:3 dilution. W and CPC groups had a more significant effect on *S. mutans* biofilm.

**Clinical relevance:**

*S. mutans* is an important bacterium in dental caries and periodontal diseases. Our study showed that non-fluoridated mouthwashes affect the initial stages of biofilm formation.

**Supplementary Information:**

The online version contains supplementary material available at 10.1007/s00784-025-06462-7.

## Introduction

Mouthwashes are an important adjunct to oral hygiene, with many different products being commercially available worldwide [1]. Mouthwashes are a heavily utilized oral care vehicle, with about 200 million mouthwash users in the United States alone [2]. These products can be classified based on their purpose into either therapeutic or cosmetic mouthwashes. Therapeutic mouthwashes are available both over the counter and by prescription, depending on the type or concentration of the active ingredient, and help to control biofilm (dental plaque), gingivitis, bad breath, dental caries [3], and dental erosion [4]. Cosmetic products intend to cleanse, beautify, promote attractiveness, or alter the appearance without the presence of “drugs” that have therapeutic purposes. Furthermore, some products may not fall under one definition or the other. Therefore, another consideration in classifying a product is the intended use of the product, which is largely dependent on the claims made for the product and the accompanying labeling.

Most products have the objective of controlling dental plaque with the prevention and treatment of caries or gingivitis. In the United States, caries prevention can only be claimed for mouthwashes containing sodium fluoride, whereas a wider range of active ingredients can be utilized to claim gingivitis prevention. Many ingredients have been evaluated for their plaque-reducing effectiveness and ability to reduce *Streptococcus mutans*, including chlorhexidine, essential oils, triclosan, cetylpyridinium chloride, sodium dodecyl sulfate, and various metal ions (tin, zinc, copper) [5]. However, the evidence supporting the effectiveness of antiplaque agents in preventing dental caries, except for chlorhexidine, is very limited [6].

“Chlorhexidine has long been regarded as a benchmark oral antimicrobial due to its proven efficacy; however, recent evidence has raised concerns regarding safety and the emergence of resistant strains, prompting re-evaluation of its routine use.” [6, 7]. Mouthwashes containing essential oils or cetylpyridinium chloride have also been extensively studied; however, there is controversy regarding their effectiveness in preventing gingivitis [8–10]. These and other ingredients have been shown to reduce the accumulation of dental biofilm [11, 12]. Mouthwashes can suppress or reduce bacterial load; however, they are intended to suppress bacterial adhesion during the initial stages of dental biofilm formation and not for mature biofilms [13].

Although *S. mutans* is not a primary colonizer in dental biofilm, it plays a key role in the progression of dental caries under conditions that favor acidogenic and aciduric microorganisms. The effectiveness of antimicrobial mouthwashes against *S. mutans* is particularly relevant during early biofilm development; however, it is important to recognize that initial enamel demineralization often involves other streptococcal species, not *S. mutans* alone [13–15]. These mouthwashes act through various mechanisms: some primarily cause membrane disruption [7], while others combine membrane disruption with the inactivation of essential enzymes [8], impairing nutrient transport across the cell wall [16] and leading to additional cellular effects. Regardless of the specific mechanism, all aim to exert a bactericidal effect.

In recent years, a substantial number of fluoride-free mouthwashes [17] have entered the market, promoted for their antimicrobial, whitening, or cosmetic benefits. Unlike fluoride-containing mouthwashes, which primarily aim to prevent dental caries through enamel remineralization, fluoride-free products often utilize alternative active agents such as cetylpyridinium chloride, essential oils, and hydrogen peroxide to reduce bacterial load or enhance oral hygiene. However, these products cannot claim caries prevention under U.S. regulatory standards, despite being widely available to consumers. Given the lack of scientific evaluation of their antimicrobial effects against cariogenic bacteria such as *S. mutans*, it is critical to investigate their efficacy, particularly at early stages of biofilm development. Therefore, this study focused specifically on fluoride-free mouthwashes to fill this gap in the literature. Hence, the present in vitro study aimed to evaluate the efficacy of commercially available, fluoride-free mouthwashes sold in Indianapolis, IN, on initial *S. mutans* biofilm.

The central hypothesis of this study is that fluoride-free commercially available mouthwashes differ in their antimicrobial efficacy against *S. mutans*, and that specific active ingredients such as cetylpyridinium chloride (CPC) and whitening agents (e.g., hydrogen peroxide, sodium hexametaphosphate) exert significantly greater inhibitory effects on *S. mutans* planktonic growth, bactericidal activity, and early biofilm formation compared to other fluoride-free formulations containing essential oils, natural-derived actives (NDA), zinc chloride, or undefined components.

## Material and methods

### Experimental design

An in vitro study was performed using eighty-one different types of previously identified fluoride-free mouthwash sold in Indianapolis, IN, USA (Appendix 1). A 16-h culture of *S. mutans* UA159 (ATCC 700610) in sterile 96-well microtiter plates was treated with the mouthwashes in three different dilutions (1:3, 1:6, and 1:12), prepared in tryptic soy broth (TSB) supplemented with 1% sucrose (TSBS) approximately 10^6^ colony- forming units. Two controls were used, chlorhexidine as a positive control and TSB like a negative control. The minimum inhibitory concentrations (MIC); (595 nm), planktonic (490 nm), and biofilm growth (490 nm) were evaluated using a spectrophotometer (SpectraMax 190; Molecular Devices, CA, USA). In addition, the minimum bactericidal concentration (MBC) growth was evaluated using five μL of each culture incubated for 48 h on blood agar. The mouthwashes were separated into six groups according to their active ingredients to analyze the results. Additional confocal laser scanning microscopy was performed to confirm the different effects.

### Mouthwash selection

This in vitro study tested 81 commercially available fluoride-free mouthwashes (Appendix 1). The products were selected to represent a broad range of formulations available over-the-counter in the local market and were grouped based on the labeled active ingredient(s): cetylpyridinium chloride (CPC; n = 25), essential oils (EO; n = 10), whitening agents (W; n = 12, containing hydrogen peroxide or sodium hexametaphosphate), Natural-Derived Actives (NDA; n = 15), zinc chloride (ZC; n = 3), and others (O; n = 16) (Table 1). The rationale for this grouping was based on the primary listed antimicrobial or cosmetic agent. Products with multi-component or ambiguous labeling were categorized into the"Others"group. Grouping was performed a priori to facilitate comparisons by formulation class.

**Table 1:** Classification of Fluoride-Free Mouthwashes by Primary Active Ingredient Group

Code		Name		Active Ingredient
CPC	Cetylpyridinum chloride		Cetylpyridinum chloride (with or without concentration)	
EO	Essential Oils		Eucalyptol, Menthol, Methyl Salicylate and Thymol (with or without concentration)	
W	Whitening		Hydrogen Peroxide, Sodium hexametaphosphate (with or without concentration)	
NDA	Natural-Derived Actives		Plant extracts, sea salt, and charcoal as natural-origin ingredients	
ZC	Zinc Chloride		Zinc chloride	
O	Other		Other ingredients not possible to classify in the other groups	

Mouthwashes were grouped based on the primary active ingredient(s) listed on the product label. Each group includes products with or without specified concentrations. “Other” refers to formulations that could not be classified into any of the defined categories due to incomplete or ambiguous labeling

### MIC, MBC, planktonic, and biofilm

A 16-h culture of *Streptococcus mutans* UA159 (ATCC 700610) was prepared in tryptic soy broth (TSB) and incubated at 37 °C under 5% CO_2_. Serial dilutions of each mouthwash (1:3, 1:6, and 1:12, prepared in TSB supplemented with 1% sucrose [TSBS]) were prepared prior to testing. In sterile, flat-bottom 96-well microtiter plates (Fisher Scientific, Newark, DE, USA), 10 μL of the standardized *S. mutans* suspension was added to 190 μL of each mouthwash dilution. The plates were then incubated at 37 °C, 5% CO_2_ for 16 h.

The minimum inhibitory concentration (MIC) was defined as the lowest dilution showing visible inhibition of bacterial growth, determined visually and confirmed by measuring absorbance at 595 nm using a microplate spectrophotometer (SpectraMax 190; Molecular Devices, CA, USA). Following MIC determination, 120 μL of the supernatant from each well was carefully transferred to a new microplate to assess planktonic growth separately from adherent bacteria. Planktonic growth was measured by optical density at 490 nm, allowing for more specific evaluation of non-adherent cells.

For MBC determination, 5 µL from each well was spot-plated onto blood agar and incubated for 48 h under the same conditions. Colony absence indicated bactericidal effect. To assess biofilm, wells were washed, fixed with 10% formaldehyde, stained with 0.5% crystal violet, rinsed, and decolorized with 2-isopropanol. Absorbance was measured at 490 nm. Potential colorimetric interference from strongly pigmented products was considered and discussed in the limitations[18].

### Confocal laser scanning microscopy

A 16-h culture of *S. mutans* UA159 was grown in TSB at 37 °C in 5% CO_2_. One mouthwash with a low-growth inhibitory effect and one with a higher effect were selected for the analysis (Crest bacteria blast mouthwash: high effect and Sea salt oral rinse: low effect), also the negative control, positive control and the medium TSBS were analyzed. Bacterial biofilms were stained with the LIVE/DEAD™ BacLight™ Bacterial Viability Kit (Invitrogen, Thermo Fisher Scientific, MA, USA), using SYTO 9 and propidium iodide (PI)LIVE/DEAD™ BacLight™ Bacterial Viability Kit (Invitrogen, Thermo Fisher Scientific, MA, USA), using SYTO 9 and propidium iodide (PI). Fluorescent images were obtained by an Olympus FV1000-MPE Confocal/Multiphoton microscope (Olympus Corp., USA) with Olympus FV10-ASW software (Olympus Corp., USA) in the Division of Nephrology, Indiana Center for Biological Microscopy, Indiana University School of Medicine. The Alexa Fluor 488 and Alexa Fluor 647 fluorescent channels were selected to detect green and red fluorescence, respectively. The image processing and analysis program was written according to Heydorn’s principles19by MATLAB1R2012 (The MathWorks, Inc.) via the codes reported before [18]

### Statistical analysis

All experiments were performed in quadruplicate and repeated three times independently. For MIC, planktonic, and biofilm data, two-way ANOVA was conducted using SAS version 9.4 (SAS Institute Inc., Cary, NC, USA), followed by Tukey’s post-hoc test. The two fixed factors included (1) mouthwash group (6 levels) and (2) dilution (3 levels). Normality and homogeneity of variance were tested prior to analysis. MBC was analyzed descriptively and categorized by minimum dilution showing bactericidal effect (< 1:3, < 1:6, < 1:12, > 1:12). The sampling unit was the individual mouthwash, and the biological replicate was the average of triplicate experiments per product. Significance was set at p < 0.05.

## Results

Of the 81 identified types of mouthwash, 63% (n = 51) were considered cosmetic, and 37% (n = 30) were therapeutic, with 9% (n = 7) intended to be used prior to rather than after toothbrushing. The major active ingredients found in the mouthwashes were CPC (n = 25), EO (n = 10), W (n = 12), ZC (n = 3), NDA (n = 15), and O (n = 16). The declared CPC concentrations ranged from 0.05% to 0.1% (9 products did not state a concentration). Nine of the ten mouthwashes containing EO declared the concentration of EO (Eucalyptol; 0.092%, Menthol 0.042%, Methyl Salicylate 0.060%, and Thymol 0.064%), with all containing alcohol (21.6–26.9 vol.%). None of the ZC mouthwashes declared the ZC concentrations. NDA mouthwashes presented a great variety of ingredients.. Of all the mouthwashes, 32% contained alcohol in concentrations of 4.1—26.9%, and four products listed alcohol as an ingredient but did not declare its concentration. Alcohol-containing mouthwashes were associated with the following main purposes: antiplaque/anti-gingivitis (33.3%) effects, management of bad breath (24.7%), and whitening (9.9%).

The results of the statistical analyses performed to compare the effects of mouthwash exposure (between the dilution of each mouthwash) and their interaction on MIC, planktonic, and biofilm, and MBC and percentage of reduction of values compared with the negative control, and the statistical difference in the first dilution (1:3) of each mouthwash with each other for the MIC and biofilm analyses are provided in appendices 2 and 3.

### Minimum inhibitory concentrations

For the MIC (Table 2) analysis, the negative control provided an absorbance value of 0.538 ± 0.052 (mean ± standard deviation). For the mouthwashes, absorbance values between 0.023–0.281, 0.0669–0.229, and 0.074–0.1972 were found for the 1:3, 1:6, and 1:12 dilutions, respectively. W mouthwashes presented the statistically significantly greatest inhibitory effect in the 1:3 dilution. CPC mouthwashes displayed the second-best inhibitory effect. These mouthwashes presented a difference from all other groups except ZC mouthwashes. EO mouthwashes were the third most effective, being superior to all. The EO group presented a statistical difference between all groups except group O. The NDA, ZC, and O groups presented a smaller inhibitory effect for the W, CPC and EO group; no statistical difference was found with the O group. And for ZC only, there was no statistical difference with the CPC group.

**Table 2:** Mean Optical Density (OD595 ± SD) and p-values for Pairwise Comparisons of *S. mutans* minimum inhibitory concentrations Among Mouthwash Groups at Three Dilutions (1:3, 1:6, and 1:12) (CPC: cetylpyridinium chloride; EO: Essential Oils; W: whitening; NDA; Natural-Derived Actives; ZC: zinc chloride; and O: Others)

Group/MIC	1:3							1:6								1:12						
	**Mean; SD**	**CPC**	**EO**	**W**	**NDA**	**ZC**	**O**	**Mean; SD**	**CPC**	**EO**	**W**	**NDA**	**ZC**	**O**	**Mean; SD**		**CPC**	**CPC**	**EO**	**W**	**NDA**	**ZC**
**Negative Control**	0.5384 ± 0.0518	<.0001	<.0001	<.0001	<.0001	<.0001	<.0001	0.5384 ± 0.0518	<.0001	<.0001	<.0001	<.0001	<.0001	<.0001	0.5384 ± 0.0518		<.0001	<.0001	<.0001	<.0001	<.0001	<.0001
**CPC**	0.0455 ± 0.0625	-	<.0001	<.0001	<.0001	0.6481	<.0001	0.0815 ± 0.0833	-	<.0001	<.0001	<.0001	0.021	<.0001	0.0736 ± 0.0700		-	<.0001	<.0001	<.0001	0.0042	<.0001
**EO**	0.0637 ± 0.0829	-	-	<.0001	0.0023	0.0107	0.3728	0.2530 ± 0.0860	-	-	<.0001	<.0001	0.0001	0.0354	0.3904 ± 0.0517		-	-	<.0001	<.0001	<.0001	<.0001
**W**	0.0082 ± 0.0129	-	-	-	<.0001	0.0196	<.0001	0.0101 ± 0.0114	-	-	-	<.0001	<.0001	<.0001	0.0123 ± 0.0124		-	-	-	<.0001	<.0001	<.0001
**NDA**	0.1101 ± 0.1056	-	-	-	-	0.0196	0.3728	0.1824 ± 0.1194	-	-	-	-	0.0037	0.234	0.2492 ± 0.1198		-	-	-	-	0.0022	0.1143
**ZC**	0.1073 ± 0.1516	-	-	-	-	-	0.0316	0.1278 ± 0.1661	-	-	-	-	-	0.0001	0.1558 ± 0.1460		-	-	-	-	-	0.0042
**O**	0.1054 ± 0.1076	-	-	-	-	-	-	0.2043 ± 0.1589	-	-	-	-	-	-	0.2579 ± 0.1558		-	-	-	-	-	-

### Planktonic

Analysis of planktonic growth revealed significant differences in antimicrobial activity among the six mouthwash groups (Table 3). W and CPC group mouthwashes exhibited the greatest inhibitory effects, showing statistically significant reductions in optical density (OD490) across all tested dilutions when compared to the negative control (p < 0.001). The EO group showed a moderate reduction in planktonic growth, with variable responses depending on the product and dilution. In contrast, the NDA, ZC, and O groups demonstrated lower and more inconsistent inhibition of *S. mutans* planktonic cells, with several products showing little to no effect. These findings highlight that W and CPC formulations were the most effective in reducing free-floating bacterial populations under the tested conditions.

**Table 3:** Mean Optical Density (OD490 ± SD) and p-values for Pairwise Comparisons of Planktonic Growth of *S. mutans* Among Fluoride-Free Mouthwash Groups at Three Dilutions (1:3, 1:6, and 1:12) (CPC: cetylpyridinium chloride; EO: Essential Oils; W: whitening; NDA; Natural-Derived Actives; ZC: zinc chloride; and O: Others)

Group/Planktonic	1:3							1:6								1:12						
	**Mean; SD**	**CPC**	**EO**	**W**	**NDA**	**ZC**	**O**	**Mean; SD**	**CPC**	**E**	**W**	**NDA**	**ZC**	**O**	**Mean; SD**		**CPC**	**CPC**	**EO**	**W**	**NDA**	**ZC**
**Negative Control**	0.1296 ± 0.0750	<.0001	<.0001	<.0001	<.0001	<.0001	<.0001	0.1296 ± 0.0750	<.0001	<.0001	<.0001	<.0001	<.0001	<.0001	0.1296 ± 0.0750		<.0001	<.0001	<.0001	<.0001	<.0001	<.0001
**CPC**	0.2841 ± 0.5149	-	0.0005	<.0001	<.0001	<.0001	<.0001	0.2293 ± 0.4795	-	0.0004	<.0001	<.0001	0.0007	0.0037	0.1972 ± 0.4543		-	0.0012	<.0001	<.0001	0.0061	0.0005
**EO**	0.0731 ± 0.2550	-	-	<.0001	<.0001	<.0001	<.0001	0.1505 ± 0.3433	-	-	<.0001	<.0001	<.0001	<.0001	0.1544 ± 0.4101		-	-	<.0001	<.0001	<.0001	<.0001
**W**	0.2103 ± 0.4921	-	-	-	0.9649	0.7305	0.0024	0.1299 ± 0.3867	-	-	-	<.0001	0.038	<.0001	0.1666 ± 0.4411		-	-	-	0.0005	0.0017	<.0001
**NDA**	0.0997 ± 0.3214	-	-	-	-	0.6042	0.0004	0.1210 ± 0.3686	-	-	-	-	0.5192	0.001	0.0743 ± 0.2870		-	-	-	-	0.5845	<.0001
**ZC**	0.0231 ± 0.0644	-	-	-	-	-	0.1013	0.0669 ± 0.1887	-	-	-	-	-	0.015	0.1722 ± 0.5262		-	-	-	-	-	0.0285
**O**	0.1306 ± 0.3920	-	-	-	-	-	-	0.1541 ± 0.4218	-	-	-	-	-	-	0.0800 ± 0.2594		-	-	-	-	-	-

### Biofilms

For the inhibition of biofilm results (Table 4), the negative control provided an absorbance of 0.799 ± 0.185. The best inhibitory result in the 1:3 dilution for the mouthwashes was for the W group; only, it did not present a statistical difference with the ZC group. They did not present a statistical difference between the ZC and EO groups. The EOgroup did not present a statistical difference between the ZC and CPC groups. The NDA group presented a statistical difference from the ZC and O groups. The O group and the negative control presented statistical differences between all the other groups and between them.

**Table 4:** Mean Biofilm Absorbance (OD490 ± SD) and p-values for Pairwise Comparisons of *S. mutans* Biofilm Formation Among Fluoride-Free Mouthwash Groups at Three Dilutions (1:3, 1:6, and 1:12) (CPC: cetylpyridinium chloride; EO: Essential Oils; W: whitening; NDA; Natural-Derived Actives; ZC: zinc chloride; and O: Others)

Group/Biofilm	1:3							1:6							1:12						
Group	**Mean; SD**	**CPC**	**EO**	**W**	**NDA**	**ZC**	**O**	**Mean; SD**	**CPC**	**EO**	**W**	**NDA**	**ZC**	**O**	**Mean; SD**	**CPC**	**EO**	**W**	**NDA**	**ZC**	**O**
Negative Control	0.7994 ± 0.1849	<.0001	<.0001	<.0001	<.0001	<.0001	<.0001	0.7994 ± 0.1849	<.0001	<.0001	<.0001	<.0001	<.0001	<.0001	0.7994 ± 0.1849	<.0001	<.0001	<.0001	<.0001	<.0001	<.0001
CPC	0.0447 ± 0.0140	-	0.958	0.01	<.0001	0.5449	<.0001	0.0727 ± 0.1482	-	<.0001	<.0001	<.0001	0.0912	<.0001	0.0698 ± 0.1726	-	<.0001	<.0001	<.0001	<.0001	<.0001
EO	0.0638 ± 0.0090	-	-	0.031	<.0001	0.2756	<.0001	0.4053 ± 0.2165	-	-	<.0001	0.2606	0.0006	0.1041	0.6756 ± 0.2643	-	-	<.0001	<.0001	<.0001	<.0001
W	0.0194 ± 0.0040	-	-	-	<.0001	0.8833	<.0001	0.0162 ± 0.0214	-	-	-	<.0001	0.383	<.0001	0.0263 ± 0.0508	-	-	-	<.0001	<.0001	<.0001
NDA	0.1953 ± 0.1190	-	-	-	-	0.0144	0.067	0.3576 ± 0.2352	-	-	-	-	0.0015	0.4537	0.4995 ± 0.2685	-	-	-	-	0.0014	0.1237
ZC	0.2253 ± 0.000	-	-	-	-	-	0.067	0.2492 ± 0.3571	-	-	-	-	-	0.0183	0.3004 ± 0.3013	-	-	-	-	-	0.0708
O	0.1795 ± 0.2029	-	-	-	-	-	-	0.3531 ± 0.3106	-	-	-	-	-	-	0.4699 ± 0.3447	-	-	-	-	-	-

### Minimum bactericidal concentrations

The MBC results showed that the majority W and CPC-containing mouthwashes exhibited strong bactericidal activity against *S. mutans*, as evidenced by the need for dilutions greater than 1:12 to observe bacterial growth. For the EO group, most mouthwashes required a dilution between 1:3 and 1:6 to inhibit bacterial growth completely. The NDA, ZC, and O groups exhibited greater variability, with some products achieving bactericidal effects at 1:3 dilution and others demonstrating limited effectiveness even at lower dilutions. These findings reinforce the higher antimicrobial potential of W and CPC groups compared to the other formulations tested. Detailed results for each product are presented in Table 5.

**Table 5:** Distribution of Minimum Bactericidal Concentration (MBC) Values Among Fluoride-Free Mouthwash Products Grouped by Active Ingredient (CPC: cetylpyridinium chloride; EO: Essential Oils; W: whitening; NDA; Natural-Derived Actives; ZC: zinc chloride; and O: Others)

CPC		EO		W		NDA		ZC		O	
Code	MBC	Code	MBC	Code	MBC	Code	MBC	Code	MBC	Code	MBC
R	> 1:12	BM	< 1:3	BJ	> 1:12	AW	< 1:3	CA	> 1:12	BF	> 1:12
E	> 1:12	O	< 1:3	BD	> 1:12	BT	> 1:12	BZ	> 1:12	H	< 1:3
G	> 1:12	BP	< 1:3	BE	> 1:12	U	> 1:12	AE	< 1:3	BL	> 1:12
V	> 1:12	I	< 1:3	BH	> 1:12	AU	< 1:6			K	> 1:12
Y	> 1:12	BQ	< 1:6	AY	> 1:12	M	< 1:6			AZ	< 1:3
F	> 1:12	AB	< 1:3	C	> 1:12	Q	< 1:3			BK	< 1:3
CB	> 1:12	AI	< 1:3	BU	> 1:12	AM	< 1:3			BG	> 1:12
CC	> 1:12	AL	< 1:3	BR	> 1:12	AK	< 1:3			J	< 1:6
BS	> 1:12	BO	< 1:3	BC	> 1:12	T	< 1:3			AG	> 1:12
AR	> 1:12	AD	< 1:3	X	> 1:12	AX	< 1:3			AA	< 1:3
AC	> 1:12			D	> 1:12	BA	< 1:3			L	< 1:6
AQ	> 1:12			AJ	> 1:12	AT	< 1:3			W	< 1:3
BV	> 1:12					AV	< 1:3			BB	< 1:3
BI	> 1:12					BY	< 1:3			S	< 1:3
Z	> 1:12					BW	< 1:3			BX	< 1:3
A	> 1:12									B	< 1:3
P	> 1:12										
AN	> 1:12										
AO	> 1:12										
N	> 1:12										
AP	> 1:12										
BN	> 1:12										
AS	> 1:12										
AH	> 1:12										
AF	< 1:3										

^**^MBC values represent the lowest dilution at which no bacterial growth was observed. *MBC values* > *1:12* indicate that the mouthwash was bactericidal at high dilution. *MBC values* < *1:3* indicate a lower bactericidal effect, requiring a more concentrated formulation for bacterial inhibition

**Fig. 1:**
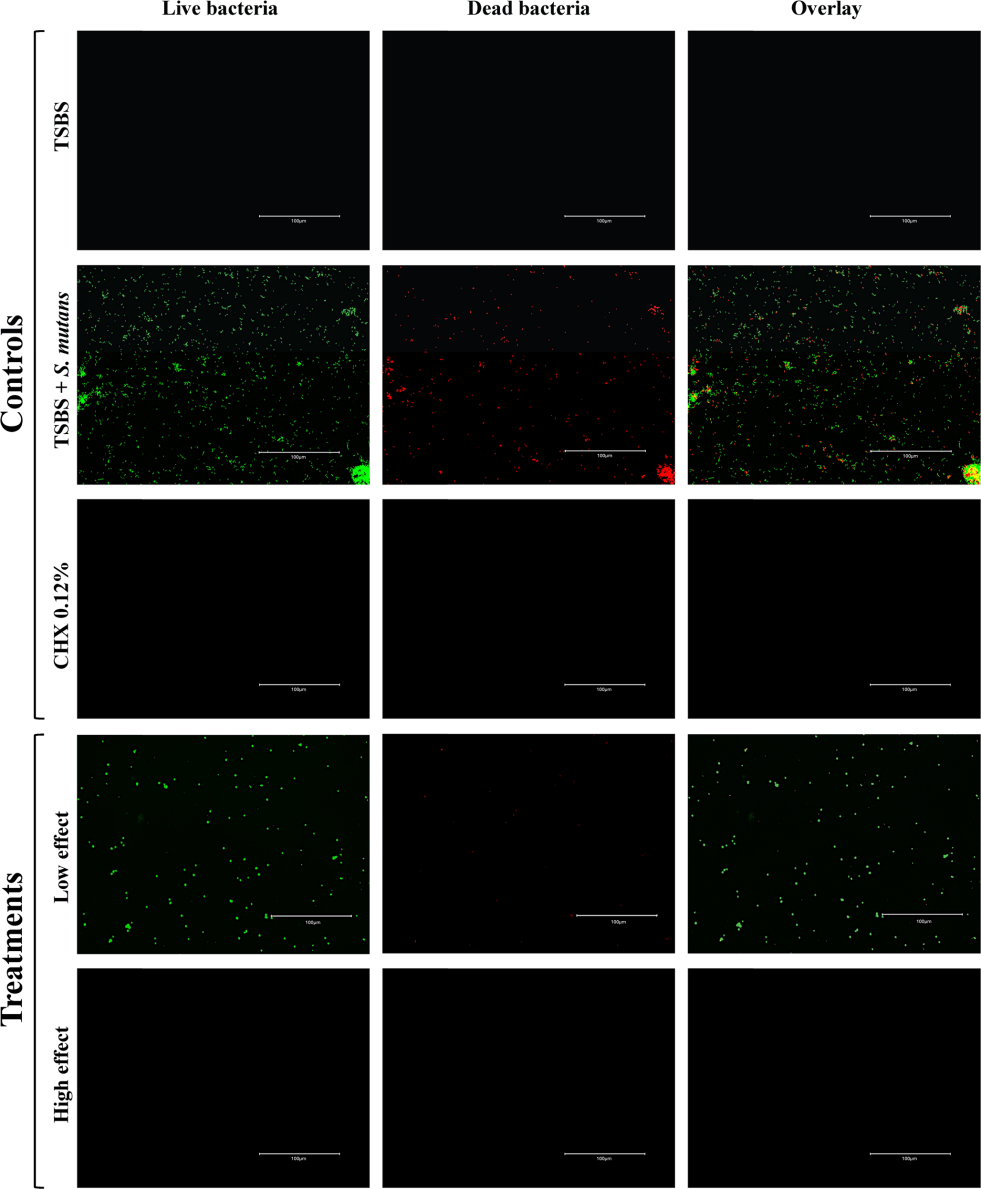
Confocal laser scanning microscopy for comparison between controls (only medium; TSBS, negative control; medium + bacteria; TSBS + S. mutans, positive control; CHX0.12%) and treatments: Crest bacteria blast mouthwash: high effect and Sea salt oral rinse: low effect. (green: vital/red: non-vital)

The confocal analysis confirmed the antimicrobial effect of the mouthwashes. The mouthwash that had a significant antimicrobial effect was used to examine the number of live bacteria, and this treatment demonstrated very few live (green) bacteria (BC Crest bacteria blast mouthwash: high effect). In addition, a second mouthwash was used to demonstrate low antimicrobial activity (B Sea salt oral rinse: low effect) (Fig. 1).

In the appendices 2 and 3, Whitening and CPC mouthwashes demonstrated the most consistent and significant reductions in *S. mutans* planktonic growth and biofilm formation across all tested dilutions, with minimum bactericidal concentrations (MBC) effective beyond a 1:12 dilution. Essential oils showed moderate efficacy, with bactericidal activity typically between 1:3 and 1:6. In contrast, products grouped under NDA, ZC, and O displayed greater variability in antimicrobial performance, with several formulations failing to achieve consistent inhibition or bactericidal effects. These observations suggest that the active ingredient's type and consistency significantly influence antimicrobial outcomes, further supporting the need for clearer labeling and standardized formulation practices.

## Discussion

This in vitro study evaluated the antimicrobial effects of fluoride-free mouthwashes on *Streptococcus mutans* using planktonic growth inhibition, minimum inhibitory concentration (MIC), minimum bactericidal concentration (MBC), and early-stage biofilm disruption assays. The decision to investigate fluoride-free formulations reflects their growing commercial availability and consumer preference for"natural"or cosmetic products. These products often claim antibacterial or breath-freshening properties but lack fluoride, a well-established agent for remineralization and caries prevention. Thus, evaluating their antimicrobial performance against *S. mutans*, a key acidogenic species in caries development, is clinically relevant.

Unlike fluoride-containing products that primarily strengthen enamel through remineralization, fluoride-free mouthwashes depend on other active ingredients such as CPC, EO, hydrogen peroxide (HP), and NDA to exert antimicrobial effects. However, their efficacy, particularly at commercial concentrations, remains underexplored.

*S. mutans* is recognized for its role in biofilm maturation and enamel demineralization due to its acidogenicity and aciduric properties. While not a pioneer colonizer, its expansion under cariogenic conditions makes it a suitable target for antimicrobial evaluation. Nevertheless, we acknowledge the limitations of using a mono-species model, which does not fully replicate the complexity of polymicrobial oral biofilms*.* [13–16].

Chlorhexidine (CHX) at 0.12% was included as a positive control due to its long-standing use and broad-spectrum antimicrobial efficacy in oral care. However, its historical classification as a “gold standard” is increasingly debated due to associated side effects such as tooth staining, taste alteration, cytotoxicity, and genotoxicity with prolonged use [6, 19]. These limitations highlight the importance of exploring alternative agents with fewer adverse effects.

Among the tested products, whitening mouthwashes (W group) demonstrated the strongest antimicrobial effects across planktonic, MIC, MBC, and biofilm assays. This likely reflects the antimicrobial properties of hydrogen peroxide, which can damage bacterial membranes and proteins (Steinberg et al., 2020). CPC-containing mouthwashes also showed high efficacy, consistent with its known mechanism of membrane disruption and enzyme inhibition [19–21] EO-based mouthwashes produced moderate antimicrobial effects, aligning with previous studies showing variable but consistent anti-biofilm and anti-gingivitis activity [11, 22].

MBC analysis indicated that whitening and CPC-containing mouthwashes achieved bactericidal effects at higher dilutions, consistent with their MIC performance. EO-based mouthwashes were effective primarily between 1:3 and 1:6 dilutions. The NDA, ZC, and O groups presented high variability in MBC outcomes, likely due to diverse formulations and unknown concentrations. These findings agree with reports that bactericidal efficacy varies widely based on active compound concentration and delivery vehicle [23, 24]. Although the zinc chloride (ZC) group included only three products, it is worth noting that zinc has been recognized for its antimicrobial properties, primarily through its ability to interfere with bacterial glycolysis, inhibit plaque formation, and reduce bacterial adhesion. In the present study, products containing ZC showed moderate antimicrobial effects across MIC, MBC, and biofilm assays, but their performance was less consistent compared to CPC or whitening formulations. This variability may reflect differences in formulation, concentration, or pH buffering systems among ZC-containing products. Prior research suggests that zinc can enhance the action of other active agents, especially in multi-component mouthwashes; however, its standalone effectiveness remains limited and formulation-dependent [25, 26] Future investigations using well-characterized concentrations of zinc, both alone and in combination, would help clarify its specific role in oral biofilm control.

Inhibition of early biofilm formation largely mirrored planktonic and MIC results, with whitening agents again showing the strongest effects, followed by CPC and EO. Although *S. mutans* is not among the initial colonizers of dental biofilms, targeting its early adhesion and proliferation remains relevant to interrupt biofilm maturation and acid production in caries-prone individuals.

Alcohol was present in approximately one-third of the mouthwashes. While alcohol may enhance solubility and penetration of active agents, it can also act as an independent antimicrobial factor, potentially confounding efficacy assessments, particularly in EO-containing formulations.

A major limitation of this study was the absence of declared concentrations for most active ingredients, limiting dose–response comparisons and complicating interpretation. Lack of transparency on commercial labels hinders both research evaluation and consumer decision-making. Regulatory agencies, such as the ADA, should consider mandating full disclosure of active agent concentrations in mouthwash formulations.

Other limitations include the use of a mono-species biofilm model that does not reflect the complexity of polymicrobial dental biofilms. Furthermore, while absorbance-based assays allowed for high-throughput evaluation, the presence of dyes, pigments, and formulation turbidity may have introduced optical interference. The NDA and O groups, which included highly diverse formulations, also displayed elevated variability, limiting statistical power. Moreover, only three dilutions were tested, which may not fully simulate intraoral dilution conditions. Bacterial viability was assessed via optical density without confirmatory CFU counts. Although CLSM imaging was performed to visualize early biofilm formation, quantitative live/dead cell analysis was not conducted. Future studies incorporating quantitative CLSM analysis, CFU counts, and alternative imaging techniques such as SEM are warranted to provide more robust assessments of antimicrobial effects.

Finally, we emphasize the need for future studies to include multispecies biofilm models, broader dilution ranges, and stratified comparisons between alcohol-containing and alcohol-free products. Improved regulatory oversight requiring full disclosure of active ingredient concentrations would greatly enhance the reproducibility, relevance, and clinical interpretation of antimicrobial efficacy studies in oral care products.

## Conclusions

Under the limitations of this in vitro study, fluoride-free mouthwashes exhibited variable antimicrobial effects against *S. mutans* in both planktonic and early biofilm states. The study confirmed the initial hypothesis that certain fluoride-free formulations, particularly those containing cetylpyridinium chloride and whitening agents (e.g., hydrogen peroxide or sodium hexametaphosphate), have a stronger inhibitory effect compared to other formulations. These groups consistently demonstrated reduced bacterial growth and biofilm formation across tested dilutions, while products containing essential oils, natural-derived actives, zinc chloride, or other agents showed more limited or inconsistent activity. These findings highlight that not all fluoride-free mouthwashes possess equal antimicrobial potential, emphasizing the need for more transparent labeling, further in situ or clinical validation, and careful consideration by both clinicians and consumers when selecting fluoride-free alternatives for caries prevention.

## Supplementary Information

Below is the link to the electronic supplementary material.

Supplementary file1 (DOCX 34 KB)

Supplementary file2 (DOCX 65 KB)

Supplementary file3 (DOCX 28 KB)

## Data Availability

No datasets were generated or analysed during the current study.
