# Supplementary material for: In vitro effect of fluoride-free mouthwashes on Streptococcus mutans biofilm
Source: Clin Oral Investig. 2025 Aug 14;29(9):413. doi: 10.1007/s00784-025-06462-7 (PMC12354505; doi:10.1007/s00784-025-06462-7)
Supplement: Supplementary file 1 — Supplementary file1 (DOCX 34 KB) [file 784_2025_6462_MOESM1_ESM.docx]

**Appendix 1:** Complete information of the mouthwash samples (ingredients and purpose).

| **Code** | **Name** | **Ingredients** | | **Alcohol** | **Purpouse** |
| --- | --- | --- | --- | --- | --- |
|  |  | **Active** | **Other** |  |  |
| A | Close.UP Mouthwash with Calcium cinnamon | Cetylpyridinium Chloride, zinc gluconate, calcium lactate | Purified water, glycerin, poloxamer 407, sodium benzoate, xylitol, flavor, sodium saccharin, menthol, cetylpiridinium chloride, zinc gluconate, citric acid, calcium lactate, D&C red 33, FD&C Red 40 | _ | Antiplaque Antigingivitis |
| B | Sea salt oral rinse |  | Water, sea salt, xylitol, natural flavor, lysozyme, menthol, potassium sorbate, sodium benzoate, poloxamer 407 | _ |  |
| C | Colgate Optic White High Impact White Advanced | Hydrogen Peroxide | Water, Glycerin, Propylene Glycol, Sorbitol, Hydrogen Peroxide, Polysorbate 20, Sodium Acrylates/Methacryloylethyl Phosphate Copolymer, Phosphoric Acid, Citric Acid, Flavor, PVM/MA Copolymer, Sodium Saccharin. | _ | Bad Breath |
| D | Colgate Peroxyl | Hydrogen peroxide 1.5% /W/v) | Water, sorbitol, propylene glycol, poloxamer 338, polysorbate 20, flavor, sodium saccharin, FD&C blue no.1 | _ | Antiplaque Antigingivitis |
| E | Oral B Gum Detoxify | Cetylpyridinium Chloride 0.07%, zinc lactate | Flavor, glycerin, methylparaben, poloxamer 407, propylparaben, sodium saccharin, sucralose, water, zinc lactate | _ | Sensibility |
| F | Crest Pro-Health Bacteria Guard | Cetylpyridinium Chloride 0.05% | Water, alcohol (15wt%), propylene glycol, flavor, poloxamer 407, sucralose, benzoic acid, sodium benzoate, blue 1, yellow 6 | Alcohol 15% wt% | Prebrushing |
| G | Crest Pro-Health Clinical | Cetylpyridinium Chloride 0.1%, Hydrogen peroxide | Water, glycerin, Hydrogen peroxide, flavor, sucralose, poloxamer 407 | _ | Antiplaque Antigingivitis |
| H | Ultimate Essential MouthCare. Eco dent | Sodium Bicarbonate 2.5% | Purified water, glycerin,polysorbate 20, aloe Barbadensis leaf juice, menthol, mentha piperita oil, stevia rebaudiana extract, echinacea purpurea extract, citrus aurantium dulcis oil, citrus limon oil, lavandula angustifolia oil, ricinus communis seed oil, hydrastis canadensis leaf extract, pimpinella anisum fruit oil, pelargonium graveolens oil, rosmarinus officialis leaf/stem oil, hamamelis virginiana water, citrus aurantium bergamia peel oil, citrus paradisi seed extract, foeniculum vulgare oil, eugenia carypophyllus flower oil, ocimum basilicum oil, rosewater concentrate, ubiquinone | _ | Oral debriding agent/oral wound cleanser |
| I | Listerine Freshburst | Eucaliptol (0.092%), Menthol (0.042%), Methyl Salicylate (0.060%) Thymol (0.064%) | Water, Alcohol (21.6% v/v), sorbitol, poloxamer 407, benzoic acid, sodium saccharin, falvor, sodium benzoate, Yellow 10, green 3 | 21.6% v/v | Antiplaque Antigingivitis |
| J | Pre Brush Dental Rinse | tetrasodium pyrophosphate | Water, glycerin and/or sorbitol, alcohol 8.7%, tetrasodium pyrophosphate, benzoic acid, flavor, poloxamer 407, sodium benzoate, sodium lauryl sulfate, sodium saccharine, xanthan gum, FD&C blue nº 1, FD&C yellow nº 5 | Alcohol 8.7% | Antiplaque Antigingivitis/ bad breath |
| K | Listerin edition coconut & lime blend | _ | Water, sorbitol, propylene glycol, poloxamer 407, lauramidopropyl betaine, eucalyptol, benzoic acid, sodium benzoate, flavor, methyl salicylate, thymol, sucralose, menthol, sodium saccharin, yellow 10, green 3 | _ | Whitening mouthwash |
| L | Oral B Breath Purify | sodium chlorite | Water, Peg-40, hydrogenated castor oil, sodium chlorite, tetrasodium EDTA, sodium benzoate , sodium bicarbonate, flavor, Illicium Verum (Anise) fruit/seed oil, glycerin, sodium carbonate | _ | Antiplaque Antigingivitis |
| M | Thera Breath Dentist formulated withening fresh breath |  | Water, glycerin,polysorbate 20, sodium benzoate, PVP, Natural mint flavor, papain, D-limonene, menthol, glucose oxidase |  | Bad breath bacteria |
| N | Colgate Total Whole Mouth Health | Cetylpyridinium Chloride 0.075% | Water, glycerin, propylene glycol, sorbitol, poloxamer 407, flavor, potassium sorbate, citric acid, sodium saccharine, blue 1 | _ | Antiplaque Antigingivitis |
| O | Listerine Ultra Clean Zero alcohol | Eucaliptol, Menthol, Methyl Salicylate, Thymol | Water, sorbitol, propylene glycol, poloxamer 407, flavor, eucaliptol, zinc chloride,benzoic acid,sodium benzoate, lauramidoprpyl betaine, methyl salicylate. Thymol, menthol, sodium saccharin, sucralose,blue 1, red 33, green 3 | _ | Antiplaque Antigingivitis |
| P | Cepacol Antibacterial multi -protection mouthwash | Cetylpyridinium Chloride 0.05% | Purified water, alcohol 14% v/v, glycerin sodium phosphate dibasic, eucalyptus oil, polysorbate 80, methyl salicylate, cinnamon oil, peppermint oil, saccharin sodium, sodium phosphate monobasic anhydrous, menthol, edetate disodium , FD&C yellow #5 | Alcohol 14% | Antiplaque Antigingivitis |
| Q | Perio Brite Complete Oral Care Natural Mouthwash | _ | Water, glycerin, xylitol, Bio-Saponns (Yucca schidigera Root extract, quillaja saponaria Root extract, Dioscorea villosa (Wild Yam) Tuber Extract, Smilax aristolochiifolia Root Extract. Mentha piperita (peppermint) oil, Calendula officinalis flower extract, echinacea purpurea flower/leaf/Steam Extract, Chamomilla recutita (Matricaria), flower extract, Phytoplenolin (Centipeda cunninghamii extract), Centella asiatica Etract (Gotu Kola), Origanum vulgare Leaf oil (oregano), zanthoxylum americanum bark extract (Prickly Ash Bark), Lavandula angustifolia (Lavender) oil, Thymol, Eugenia caryophyllus (clove) bud oil, Folic Acid, Olea Europaea (Olive) Leaf extract, thymus vulgaris (Thymel) flower/Leaf oil, Eucalyptus globulus Leaf Oil, Cinnamomum zeylanicum Bark Oil, Citrus Paradisi (Grapefruit) Seed Extract, Juglans nigra (Black Walnut) Shell Extract, Ubiquinone (CoQ10), Camellia Sinensis Leaf Extract (Green Tea), Rosmarinus Officinalis (Rosemary) Leaf Oil, Aloe barbadensis Leaf juice (Aloe Vera), Citrus Linon (Lemon) Peel Oil, Hydrastis Canadensis (Goldensea) Extract, Cinnamomum cassia Bark Extract... | _ | Whitens surface of teeth |
| R | Crest Pro-Health Intense | Cetylpyridinium Chloride 0.05% | Water, alcohol (15wt%), propylene glycol, flavor, poloxamer 407, sucralose, benzoic acid, sodium benzoate, blue 1, yellow 6 | Alcohol 15% wt% | _ |
| S | Hello Activated Charcoal |  | Puridied water, vegetable glycerin (soother and moisturizes), polysorbate 80 (emulsifier), flavor (yum), xylitol (aweetener), erythritol (sweetener), poloxamer 407 (emulsifier), charcoal powder (freshens breath), cocos nucifera (coconut) oil (soothes and moisturizes), sodium benzoate (maintains stability), melaleuca alternifolia (tea tree) leaf oil (freshens breath) | _ | Bad Breath |
| T | Jason Halthy mouth Tartar Control cinnamon clove | _ | Water, glycerin, polysorbate 20, cinnamon zeylanicum bark oil, eugenia caryophyllus flower oil, melaleuca alternifolia leaf oil, melia azadirachta seed oil, Aloe barbadensis leaf juice, calendula officinalis flower extract, citrus grandis seed extract, echinacea purpurea extract, hamamelis virginiana water, hydrastis canadensis extract, melissa officinalis leaf extract, perilla ocymoides seed extract, ascorbic acid, calcium ascorbate, menthol, sodium bicarboante, sea salt, cinnamal, eugenol | _ | Whitening mouth rinse |
| U | Thera Breath Dentist formulated fresh breath | sodium chlorite | Water, Peg-40, hydrogenated castor oil, sodium chlorite, tetrasodium EDTA, sodium benzoate , sodium bicarbonate, mentha piperita (peppermint) oil, sodium hydroxide |  | Antiplaque Antigingivitis |
| V | Parodontax active gum health Mint | Cetylpyridinium Chloride 0.07% | Water, glycerin, flavor, poloxamer 188, sodium saccharin, propylene glycol, sodium benzoate, sucralose, benzoic acid | Alcohol | Dry mouth |
| W | Biotène dry mouth oral rinse | _ | Water, glycerin, xylitol, sorbitol, propylene glycol, poloxamer 407, sodium benzoate, hydroxyethylcellulose, methylparab, propylparaben, flavor, sodium phosphate, disodium phosphate | _ | Bad Breath |
| X | ARC turn up the bright | Hydrogen peroxide, sodium hexametaphosphate | Water, glycerin, propylee glycol, hydrogen peroxide, sodium hexametaphosphate, poloxamer 407, flavor, sodium citrate, sodium saccharin, citric acid, sucralose | _ | Antiplaque Antigingivitis |
| Y | Crest Pro-Health Clean Mint multiprotection | Cetylpyridinium Chloride 0.07% | Water, glycerin, flavor, poloxamer 407, sodium saccharin, methylparaben, sucralose, propylparaben, blue 1 | _ | Whitening mouth rinse |
| Z | Mouthwash up&up | Cetylpyridinium Chloride | Water, alcohol 15 wt%, glycerin, flavor, polysorbate 80, sodium saccharin, sodium benzoate, cetylpiridinium chloride, benzoic acid, blue 1, yellow 5 | Alcohol 15% wt% | Antiplaque Antigingivitis |
| AA | Dr. Tichenor's All natural Peppermint Mouthwash Concentrate | _ | Alcohol (70%), peppermint oil, arnica, sacccharum carbonate, USP purified water | Alcohol 70% | Teeth Whitening |
| AB | Antiseptic Mouthwash antigingivits antiplaque | Eucaliptol (0.092%), Menthol (0.042%), Methyl Salicylate (0.060%) Thymol (0.064%) | Water, Alcohol (21.6% v/v), sorbitol, flavor, poloxamer 407, benzoic acid, sodium saccharin, sodium benzoate, FD&C green nº3 | 21.6% v/v | Bad Breath |
| AC | Hello Peace out, plaque | Cetylpyridinium Chloride 0.075% | Water, glycerin, aloe barbadensis leaf juice, polysorbate 80, erythritol, xylitol, poloxamer 407, flavor, cocos nucifera (coconut) oil (certified organic), sodium benzoate, citric acid, rebaudiside A (certified organic), melaleuca aiternifoil (tea tree) leaf oil | _ | Antiplaque Antigingivitis |
| AD | Listerine Original | Eucaliptol (0.092%), Menthol (0.042%), Methyl Salicylate (0.060%) Thymol (0.064%) | Water, Alcohol (26.9 % v/v), poloxamer 407, benzoic acid sodium benzoate, Caramel | Alcohol 26.9 % v/v | Fresh breath |
| AE | Lavoris Freash Breath Mouthwash | zinc chloride | Water, alcohol, glycerin/sorbitol, flavor, poloxamer 407, polysorbate 20, dosium saccharin, zinc chloride, citric acid, blue 1 | Has, but does not describe the amount | Fresh breath |
| AF | Oral B Dry mouth | Cetylpyridinium Chloride | Flavor, glycerin, propylene glycol, xylitol, cellulose gum, sodium hyaluronate, poloxamer 407, sodium benzoate, cetylpyridinium chloride, benzoic acid | _ | Dry mouth brad |
| AG | Plax sofmint flavor mouthwash | tetrasodium pyrophosphate | Water, sorbitol, alcohol (8.6%), tetrasodium pyrophosphate, sodium benzoate, benzoic acid, sodium lauryl sulfate, poloxamer 407, flavor, xanthan gum, sodium saccharin, blue 1, yellow 5 | Alcohol 8.6 % v/v | Antiplaque Antigingivitis |
| AH | ARC fresh breath mouth rinse | Cetylpyridinium Chloride, zinc lactate | Water, glycerin, flavor, cetylpiridinium chloride, zinc lactate, methylparabe, sodium saccharin, sucralose, propylparabem, poloxamer 407 | _ | Antiplaque Antigingivitis |
| AI | Antiseptic Mouthwash up &up | Eucaliptol (0.092%), Menthol (0.042%), Methyl Salicylate (0.060%) Thymol (0.064%) | Water, alcohol 21.6%, sorbitol solution, flavor, poloxamer 407, benzoic acid, sodium saccharin, sodium citrate, D&C yellon nº 10, FD&C green nº 3 | 21.6% v/v | Bad breath prevention |
| AJ | Oral B Mouth Sore | Hydrogen peroxide 1.5% /W/v), sodium hexametaphosphtate | Citric acid, flavor, glycerin, poloxamer 407, propylene glycol, sodium citrate, sodium hexametaphosphtate, sodium saccharin, sucralose, water | _ | Antiplaque Antigingivitis |
| AK | Tom`s Natural Fluoride-free Wicked fresh | zinc citrate | Water, glycerin, sorbitol, Aloe barbadensis leaf juice (organic), propanediol, cylitol, natural flavor, benzoic acid, zinc citrate, menthol, sodium hydroxide | _ | Oral Pain Reliever |
| AL | Listerine Cool Mint | Eucaliptol (0.092%), Menthol (0.042%), Methyl Salicylate (0.060%) Thymol (0.064%) | Water, Alcohol (21.6% v/v), sorbitol, flavor, poloxamer 407, benzoic acid, sodium saccharin, sodium benzoate, FD&C green nº3 | 21.60% | Antiplaque Antigingivitis |
| AM | The Natural Dentist Healthy gums | Aloe Vera (20%) | Purified Wate, Vegetable Glycerin, Echinacea, Goldenseal, Calendula, Citric Acid, Polysorbate 80, Natural Flavors (contains cinnamon oil) Grapefruit seed extract, potassium citrate, copper chlorophyllin Color | _ | _ |
| AN | Thera breath periodontist formulated Healthy gums oral rinse | Cetylpyridinium Chloride 0.05% | Water, glycerin, poloxamer 407, flavor, sucralose |  | _ |
| AO | Mint dry mouth oral rinse |  | Water, glycerin, xylitol, sorbitol, propylene glycol, poloxamer 407, sodium benzoate, hydroxyethylcellulose, hydroxiaceatophenone, 1,2-hexanediol, caprylyl glycol, flavor, sodium phosphate, disodium phosphate | _ | Antiplaque Antigingivitis |
| AP | Swan Mouthwash fresh mint | Cetylpyridinium Chloride | Water, alcohol 15 wt%, glycerin, flavor, polysorbate 80, sodium saccharin, sodium benzoate, cetylpiridinium chloride, benzoic acid, blue 1, yellow 5 | 15.00% | Bad Breath (prevent) |
| AQ | Crest Scope mouthwash rince-bouche | Cetylpyridinium Chloride | Water, alcohol 13wt%, glycerin, flavor, polysorbate 80, sodium saccharin, sodium enzoate, cetylpiridinium Chloride, Benzoic Acid, blue 1 F.C.F, yellow 5/tartrazine | 13%% | Antiplaque Antigingivitis |
| AR | Family Wellness Blue mint Mouthwash | Cetylpyridinium Chloride | Water, alcohol (12.25 wt%), glycerin and/or sorbitol, polysorbate 80 and/or polysorbate 20, flavor, sodium saccharin, sodium benzoate, cetylpiridinium chloride, benzoic acid, blue 1 | 12.25% | Natural fresh |
| AS | Crest all fresh no stress Scope All day | Cetylpyridinium Chloride 0.07%, zinc lactate | Water, glycerin, flavor, zinc lactate, methylparaben, sodium saccharin, sucralose, propylparaben, poloxamer 407 | _ | Antiplaque Antigingivitis |
| AT | Desert Essence Tea Tree OilSpearmint | _ | Water, glycerin, polysorbate 80, Eco-Harvest Melaleuca Alternifolia leaf oil, Aloe barbadensis Leaf, juice, Mentha Viridis leaf oil, Hamamelis Virginiana extract, Ascorbic acid, calcium ascorbate, citric acid | _ | Oral debriding agent/oral antiseptic/ Oral pain reliever |
| AU | Jason Healthy powersmile brightening | _ | Water, glycerin, polysorbate 20, mentha piperita oil, aloe barbadensis leaf juice, calendula officinalis flower extract, carica papaya fruit extract, citrus grandis seed extract, echinacea angustifolia extract, hamamelis virginiana water, hydrastis canadensis extract, perilla ocymoides seed extract, ascorbic acid, calcium ascorbate, menthol, sea salt, sodium bicarbonate, xylitol | _ | Refreshing Mouth rinse |
| AV | Lumineux Oral essentials | _ | Organic Aloe Barbadensis (Aloe Vera) Leaf Juice, Xylitol, Purified water, dead sea salt, gautheria procumbens leaf oil, cocos nucifera oil, citrus limon peel oil, salvia officinalis oil, mentha ciridis leaf oil, Organic mentha piperita leaf oil, ocimum basilicum oil, Eugenia Caryophyllus flower Oil. | _ | Refreshing |
| AW | Jason Total Protection sea salt mouthrinse | _ | Water, sea salt, glycerin, sorbitol, mentha piperita oil, camellia sinensis leaf extract, citrus grandis seed extract, hamamelis virginiana extract, salvia officinalis leaf extract, ascorbic acid, citric acid, mentho, polysorbate 20, sodium bicarbonate, benzoic acid, potassium sorbate, sodium benzoate | _ | Proven to freshen breath |
| AX | Tea Tree therapy Mouth Wash | _ | Deionized Water, sorbitol, tea tree oil, (oil of Melaleuca alternifolia) Natural Mint flavor, citric acid, sodium citrate | _ | Help keep the mouth and teeeth refreshing clean |
| AY | HPM Hydrogen Peroxide Mouthwash | Hydrogen peroxide | Water, hydrogen Peroxide, alcohol, Thymus sephyllum (White thyme) Leaf Oil, Eucalyptol, Menthol, Natural Wintergreen flavor | Has, but does not describe the amount | Complete Oral Care |
| AZ | Bubble Gum Kid's Spry Mouth wash | Calcium Glycerophosphate | Purified Water, xylitol, vegetable glycerin, erythritol, Echinacea Purpurea, Chamomile, Olive Leaf, Marigold, Thyme, Oat Beta Glucan, Aloe Vera, Calcium Glycerophosphate, Cocamidopropyl betaine, Honeysuckle, Natural flavors, Color Stabilizer, Natural Plant Coloring | _ | Fresh and Clean |
| BA | Jason Halthy mouth Fresh breath cinnamon | _ | Water, glycerin, cinnamomum zeylanicum leaf oil, mentha piperita oil, citric acid, polysorbate 20, benzyl alcohol, potassium sorbate, sodium benzoate | _ | Fresh breath |
| BB | Swan Alcohol free Mouthwash | _ | Water, sorbitol, propylene glycol, poloxamer 407, flavor, benzoic acid, sodium benzoate, sodium saccharin, blue 1 | _ | Brushing Rinse |
| BC | Orajel 2x Mouth Sores Rinse Medicated | Hydrogen peroxide 1.5% Menthol 1% | Alcohol 4.1% (by volume), disodium EDTA, FD&C blue nº1, methyl salicylate, phosphoric acid, poloxamer 338, polysorbate 20, sodium saccharine, sorbitol, water | 4.10% | Brushing Rinse |
| BD | Crest Bacteria Blast | hydrogen peroxide, sodium hexametaphosphate | Water, glycerin, alcohol (5wt%), hydrogen peroxide, sodium hexametaphosphate, poloxamer 407, flavor, sodium citrate, sodium saccharin, citric acid, sucralose | Alcohol 5% wt% | Whitening mouthwash |
| BE | Clean mint Withening alcohol free rinse | Hydrogen peroxide, sodium hexametaphosphate | Water, glycerin, hydrogen peroxide, propylene glycol, sodium hexametaphosphate, poloxamer 407, sodium citrate, flavor, PEG-40, Hydrognated castor oil, soddium saccharine, citric acid. | _ | Clean and refresher |
| BF | Thera Breath dentist formulated fresh breath oral rinse | sodium chlorite | Water, glycerin, PEG-40, hydrogenated caster oil, citric acid, sodium hydroxide, pepper mint oil,menthol, sodium chlorite, citrus limon peel oil, sodium benzoate, sucralose, xylitol, sodium bicarbonate. | _ | Bad Breath |
| BG | Listerine Sensitivity Zero alcohol | dipotassium oxalate monohydrate | Water, sorbitol, propylene glycol, dipotassium oxalate monohydrate, flavor, phosphoric acid, ploxamer, sodium benzoate, sodium methyl cocoyl taurate, sodium laurylsulfate, sodium saccharin, sucralose | _ | Whitening mouthwash |
| BH | Splendid white whitening mouth rinse alcohol free Up&Up | Hydrogen peroxide, sodium hexametaphosphate | Water, glycerin, hydrogen peroxide, propylene glycol, sodium hexametaphosphate, poloxamer 407, sodium citrate, flavor, PEG-40, Hydrognated castor oil, sodium saccharine, citric acid. | _ | Feel clean and extra fresh |
| BI | Crest Scope Classic Classique | Cetylpyridinium Chloride | Water/EAU, alcohol (15wt%), glycerin, flavor, polysorbate 80, sodium saccharin, sodium benzoate, cetylpyridinium chloride, benzoic acid, blue 1. yellow 5 | Alcohol 15% wt% | Oral Wound Cleanse/Oral Debriding Agent |
| BJ | BR rinse. Organic Mouthwash | hydrogen peroxide | Aloe leaf juice, purified water, hydrogen peroxide, acacia, wintergreen oil, xanthan gum, peppermint oil, rosemary leaf oil, eucalyptus leaf oil, lemon peel oil, cinnamon leaf oil (organic ingredient) | _ | Fresh breath mouthwash |
| BK | Tom´s Sea Salt | zinc citrate | Water, glycerin, sorbitol, Aloe barbadensis leaf juice, propanediol, xylitol, sodium chloride, aroma/flavor (natural), zinc citrate, benzoic acid, menthol, sodium hydroxide | _ | Fresh breath |
| BL | CloSYS | stabilized chloride dioxide | Purified Water, Cloralstan (stabilized chloride dioxide), trisodium phosphate, citric acid, flavor, sucralose | _ | Brightening |
| BM | Antiseptic Mouthwash antigingivits antiplaque | Eucaliptol (0.092%), Menthol (0.042%), Methyl Salicylate (0.060%) Thymol (0.064%) | Water, Alcohol (26.9 % v/v), poloxamer 407, benzoic acid sodium benzoate, Caramel | Alcohol 26.9 % v/v | Bad breath bacteria |
| BN | Crest GUM and Breath Purify | Cetylpyridinium Chloride 0.07% | Water, glycerin, flavor, zinc lactate, methylparaben, sodium saccharin, sucralose, prpylparaben, poloxamer 407, blue1 | _ | Brushing Rinse |
| BO | Swan Antiseptic mouth rinse Original | Eucaliptol (0.092%), Menthol (0.042%), Methyl Salicylate (0.060%) Thymol (0.064%) | Water, Alcohol (26.9 % v/v), poloxamer 407, benzoic acid sodium benzoate, Caramel | 26.90% | Antiplaque Antigingivitis |
| BP | Swan Antiseptic Mouth rinse Spring Mint | Eucaliptol (0.092%), Menthol (0.042%), Methyl Salicylate (0.060%) Thymol (0.064%) | Water, Alcohol (21.6% v/v), sorbitol, flavor, poloxamer 407, benzoic acid, sodium saccharin, sodium citrate, D&C yellow nº 10, FD&C green nº3 | Alcohol 21.6% | Antiplaque Antigingivitis |
| BQ | Advanced Mouth Rinse | Eucaliptol (0.092%), Menthol (0.042%), Methyl Salicylate (0.060%) Thymol (0.064%), zinc chloride | Water, Alcohol (21.6% v/v), sorbitol, flavor, poloxamer 407, benzoic acid, zinc chloride, sodium benzoate, sucralose, sodium saccharin, green nº3 | Alcohol 21.6% | Bad Breath |
| BR | Crest 3D white brilliance | sodium hexametaphosphate | Water, glycerin, sodium hexametaphosphate, poloxamer 407, sodium benzoate, sodium lauryl sulfate, flavor, phosphoric acid, sodium saccharin, sucrallose, red 33, green 3 | _ | Antiplaque Antigingivitis |
| BS | Multi-action alcohol free. Antiseptic mouth rinse | Cetylpyridinium Chloride 0.07% | Water, glycerin, flavor, poloxamer 188, sodium saccharin, propylene glycol, sodium benzoate, sucralose, benzoic acid, blue 1 | _ | Fresh breath and Bad breath |
| BT | BR rinse. Organic Mouthwash Peppermint | hydrogen peroxide | Aloe leaf juice, purified water, hydrogen peroxide, acacia, peppermint oil, rosemary leaf oil, eucalyptus leaf oil, lemon peel oil, clove bud oil (eugenia caryophyllus), cinnamon leaf oil (organic ingredient) | _ | Bad Breath |
| BU | Guru Nanda Oxyburst Whitening Technology | hydrogen peroxide, calcium lactate | Purified water, glycerin, xylitol, sorbitol, hydrogen peroxide, sodium benzoate, aloe vera, sodium bicarbonate, Peg-40 hydrogenated castor oil, sodium lauroyl sarcosinate, sodium chloride, peppermint oil, stevia, cardamom oil, clove oil, calcium lactate, vitamin E, Vitamin D, spearmint oil, fenne oil, tea tree oil, oregano oil, jasmine oil, menthol, citric acid | _ | Antiplaque Antigingivitis |
| BV | PerCara Mouthwash & Gargle Refresh mint | cetylpiridinium chloride | Water, alcohol, sorbitol, polysorbate 20, flavor, cetylpiridinium chloride, sodium saccharine, sodium benzoate, benzoic acid, blue 1, yellow 5 | Has, but does not describe the amount | Clean and fresher breath |
| BW | Spry Oral Rinse (Dental defense system) | _ | Purified Water, Xylitol, grain alcohol (ethanol), coolmint flavor (blend of natural flavors), vegetable glycerin, calcium glycerophosphate, aloe vera, Marigold, Chamomile, Echinacea, Olive Leaf, Thyme | Has, but does not describe the amount | Antiplaque Antigingivitis |
| BX | Desert Essence Tea Tree Oil Whitening Plus Mouthwash | _ | Purified water, glycerin, polysorbate-80, Phyllostachis Bambusoides Juice, Mentha Viridis Leaf oil, Equisetum Arvense Leaf extract, Gaultheria Procumbens Leaf oil, Eco-Harves Melaleuca Alternifolia leaf oil, Zinc Citrate, Calcium absorbate, ascorbic acid, hamamelis virginiana extract, Stevia Rebaudiana Leaf/stem power | _ | Bad Breath |
| BY | Desert Essence Prebiotic Plant based brushing rinse Mint | _ | Water, glycerin, polysorbate 80, perilla pcymoides seed extract, cichorium intybus root extract, hydrolyzed pea protein, inulin, glycyrhiza glabra root extract, quillaja saponaria bark extract, quisetum arvense leaf extract, glutathione, mentha piperita flower/leaf/stem oil, mentha arvensis steam/leaf oil,mentha viridis leaf oil, anthemis nobilis flower oil, echinacea angustifolia extract, zinc citrate, salvia officinalis oil, commiphora myrha oil, calcium ascorbate, ascorbic acid, aloe barbadensis leaf juice, calndula officinalis flower oil, hydrastis canadensis extract, melaleuca alternifolia leaf oil. hamamelis virginiana extract, menthol, maltodextrin, xylitol phytic acid, citric acid | _ | Clean and fresher breath |
| BZ | Smart mouth Mouth Sore zinc activatted oral rinse | Menthol 0.2% (When mixed directed), sodium chlorite, zinc chloride | Solution 1: Purified water, sodium benzoate, sodium chlorite and benzoic acid. Solution 2: Purified water, sorbitol, poloxamer 407, propylene glycol, poloxamer 124, zinc chloride, flavor, sodium benzyl alcohol, sodium saccharin, speppermint oil, benzoic acid, sodium chloride, , D&C yellow Nº 10, FD&C blue Nº1 | _ | Antiplaque Antigingivitis |
| CA | Smart mouth Original | sodium chlorite, zinc chloride | Solution 1: Purified water, sodium benzoate, sodium chlorite and benzoic acid. Solution 2: Purified water, glycerin, poloxamer 407, propylene glycol, poloxamer 124, zinc chloride, flavor, sodium benzoate, benzoic acid, sodium saccharin, sodium chloride, benzyl alcohol, D&C yellow Nº 10, FD&C blue Nº1 | _ | Oral Care |
| CB | Smarth mouth Clinical zinc activated oral rinse | Cetylpyridinium Chloride 0.05% | Solution 1: Purified water, sodium benzoate, sodium chlorite and benzoic acid. Solution 2: Purified water, glycerin, poloxamer 407, propylene glycol, poloxamer 124, zinc chloride, flavor, sodium benzoate, benzoic acid, sodium saccharin, sodium chloride, benzyl alcohol, D&C yellow Nº 10, FD&C blue Nº1 | _ | Fresh breath & complete oral Hygiene |
| CC | Parodontax active gum health Clear mint | Cetylpyridinium Chloride 0.07% | Water, glycerin, flavor, poloxamer 188, sodium saccharin, propylene glycol, sodium benzoate, sucralose, benzoic acid | _ | Whitening mouthwash |
