# Supplementary material for: In vitro effect of fluoride-free mouthwashes on Streptococcus mutans biofilm
Source: Clin Oral Investig. 2025 Aug 14;29(9):413. doi: 10.1007/s00784-025-06462-7 (PMC12354505; doi:10.1007/s00784-025-06462-7)
Supplement: Supplementary file 2 — Supplementary file2 (DOCX 65 KB) [file 784_2025_6462_MOESM2_ESM.docx]

**Appendix 2:** One-way ANOVA statistical analyses performed to compare the effects of mouthwash exposure (between the dilution of each mouthwash) and their interaction on MIC, planktonic, and biofilm. Statistically significant differences are shaded.

|  |  | **MIC** | | | | | | | | | | | | **Planktonic** | | | | | | | | | | | | **Biofilm** | | | | | | | | | | | |
| --- | --- | --- | --- | --- | --- | --- | --- | --- | --- | --- | --- | --- | --- | --- | --- | --- | --- | --- | --- | --- | --- | --- | --- | --- | --- | --- | --- | --- | --- | --- | --- | --- | --- | --- | --- | --- | --- |
|  | **Dilution/**  **commercial name** | **1:3** | | | **1:6** | | | **1:12** | | | **p value 1:3 vs 1:6 dilution** | **p value 1:3 vs 1:12 dilution** | **p value 1:6 vs 1:12 dilution** | **1:3** | | | **1:6** | | | **1:12** | | | **p value 1:3 vs 1:6 dilution** | **p value 1:3 vs 1:12 dilution** | **p value 1:6 vs 1:12 dilution** | **1:3** | | | **1:6** | | | **1:12** | | | **p value 1:3 vs 1:6 dilution** | **p value 1:3 vs 1:12 dilution** | **p value 1:6 vs 1:12 dilution** |
| **A** | **Close.UP Mouthwash with Calcium Cinnamon** | 0.0452 | ± | 0.0122 | 0.0330 | ± | 0.0138 | 0.0266 | ± | 0.0140 | 0.0357 | 0.0336 | 0.0414 | 0.2053 | ± | 0.4310 | 0.2038 | ± | 0.4227 | 0.1147 | ± | 0.3924 | 1.0000 | 0.3223 | 0.1503 | 0.0123 | ± | 0.0103 | 0.0130 | ± | 0.0134 | 0.0109 | ± | 0.0120 | 0.9585 | 0.6553 | 0.6915 |
| **B** | **Sea Salt Oral Rinse** | 0.2740 | ± | 0.0135 | 0.3848 | ± | 0.0367 | 0.3870 | ± | 0.0339 | 0.0001 | 0.0001 | 0.6413 | 0.3744 | ± | 0.5779 | 0.3020 | ± | 0.5996 | 0.0233 | ± | 0.0527 | 0.8264 | 0.3870 | 0.7726 | 0.4469 | ± | 0.1612 | 0.6665 | ± | 0.1196 | 0.7060 | ± | 0.3293 | 0.0018 | 0.0111 | 0.2738 |
| **C** | **Colgate Optic White High Impact White Advanced** | 0.0043 | ± | 0.0088 | 0.0059 | ± | 0.0103 | 0.0067 | ± | 0.0109 | 0.5836 | 0.5530 | 1.0000 | 0.5857 | ± | 0.9150 | 0.0295 | ± | 0.1185 | 0.1112 | ± | 0.4162 | 0.1587 | 0.2880 | 0.9717 | 0.0481 | ± | 0.1188 | 0.0281 | ± | 0.0406 | 0.0436 | ± | 0.1062 | 0.7410 | 0.8994 | 0.6915 |
| **D** | **Colgate Peroxyl** | 0.0195 | ± | 0.0081 | 0.0177 | ± | 0.0110 | 0.0136 | ± | 0.0169 | 0.2768 | 0.0267 | 0.0486 | 0.2125 | ± | 0.6411 | 0.1771 | ± | 0.6193 | 0.4279 | ± | 0.7861 | 0.7441 | 0.3474 | 0.1977 | 0.0002 | ± | 0.0037 | 0.0058 | ± | 0.0054 | 0.0053 | ± | 0.0149 | 0.0215 | 0.7102 | 0.4179 |
| **E** | **Oral B Gum Detoxify** | 0.0000 | ± | 0.0000 | 0.0793 | ± | 0.0158 | 0.0502 | ± | 0.0184 | 0.0001 | 0.0001 | 0.0121 | 0.0876 | ± | 0.1954 | 0.2208 | ± | 0.4545 | 0.1441 | ± | 0.5094 | 0.5154 | 0.8201 | 0.4849 | 0.0000 | ± | 0.0000 | 0.0272 | ± | 0.0097 | 0.0149 | ± | 0.0076 | 0.0002 | 0.0008 | 0.0140 |
| **F** | **Crest Pro-Health Bacteria Guard** | 0.0041 | ± | 0.0067 | 0.0409 | ± | 0.0161 | 0.0350 | ± | 0.0152 | 0.0001 | 0.0001 | 0.0528 | 0.4562 | ± | 0.8266 | 0.4303 | ± | 0.8749 | 0.1913 | ± | 0.3527 | 0.7904 | 0.4792 | 0.6236 | 0.0000 | ± | 0.0000 | 0.0193 | ± | 0.0079 | 0.0139 | ± | 0.0089 | 0.0002 | 0.0002 | 0.1879 |
| **G** | **Crest Pro-Health Clinical** | 0.0000 | ± | 0.0000 | 0.0000 | ± | 0.0000 | 0.0762 | ± | 0.0279 | 1.0000 | 0.0001 | 0.0001 | 0.2198 | ± | 0.4959 | 0.0313 | ± | 0.0999 | 0.0265 | ± | 0.1058 | 0.5828 | 0.9027 | 0.7726 | 0.0003 | ± | 0.0012 | 0.0685 | ± | 0.0234 | 0.0278 | ± | 0.0139 | 0.0002 | 0.0002 | 0.0008 |
| **H** | **Ultimate Essential MouthCare. Eco-Dent** | 0.0067 | ± | 0.0076 | 0.2616 | ± | 0.2163 | 0.3873 | ± | 0.0821 | 0.0001 | 0.0001 | 0.2918 | -0.0070 | ± | 0.0151 | 0.0719 | ± | 0.0514 | 0.0111 | ± | 0.0058 | 0.0013 | 0.0081 | 0.0097 | 0.0198 | ± | 0.0302 | 0.3832 | ± | 0.5224 | 0.8929 | ± | 0.6409 | 0.0111 | 0.0004 | 0.1415 |
| **I** | **Listerine Freshburst** | 0.0320 | ± | 0.0080 | 0.2980 | ± | 0.0377 | 0.3693 | ± | 0.0292 | 0.0001 | 0.0001 | 0.0016 | 0.1357 | ± | 0.4105 | 0.1602 | ± | 0.3397 | 0.4318 | ± | 0.7020 | 0.0099 | 0.0167 | 0.9991 | 0.1292 | ± | 0.3945 | 0.2614 | ± | 0.1892 | 0.4700 | ± | 0.3554 | 0.0549 | 0.1564 | 0.2076 |
| **J** | **Pre Brush Dental Rinse** | 0.0458 | ± | 0.0048 | 0.0263 | ± | 0.0061 | 0.0208 | ± | 0.0078 | 0.0001 | 0.0001 | 0.0475 | 0.5856 | ± | 0.7629 | 0.5849 | ± | 0.8801 | 0.2005 | ± | 0.5941 | 0.5004 | 0.0293 | 0.1503 | 0.0033 | ± | 0.0045 | 0.0039 | ± | 0.0043 | 0.0675 | ± | 0.2198 | 0.7744 | 0.4462 | 0.3843 |
| **K** | **Listerine Limited Edition Coconut and Lime Blend** | 0.0125 | ± | 0.0143 | 0.2589 | ± | 0.0578 | 0.3582 | ± | 0.0803 | 0.0001 | 0.0001 | 0.0014 | 0.2914 | ± | 0.5600 | 0.5566 | ± | 0.8060 | 0.0245 | ± | 0.0060 | 0.8264 | 0.4531 | 0.1977 | 0.0093 | ± | 0.0062 | 0.3858 | ± | 0.0683 | 0.5283 | ± | 0.1648 | 0.0002 | 0.0002 | 0.0493 |
| **L** | **Oral B Breath Purify** | 0.1578 | ± | 0.0357 | 0.3366 | ± | 0.0718 | 0.4073 | ± | 0.0631 | 0.0006 | 0.0001 | 0.0181 | 0.0060 | ± | 0.0072 | 0.0185 | ± | 0.0214 | 0.0158 | ± | 0.0220 | 0.1400 | 0.1712 | 0.8354 | 0.3938 | ± | 0.1111 | 0.4814 | ± | 0.2217 | 0.5881 | ± | 0.1390 | 0.3456 | 0.0061 | 0.0701 |
| **M** | **Thera Breath Dentist Formulated Withening Fresh Breath** | 0.0249 | ± | 0.0127 | 0.0736 | ± | 0.0298 | 0.2650 | ± | 0.0656 | 0.0011 | 0.0001 | 0.0002 | 0.5100 | ± | 0.6388 | 0.7393 | ± | 0.9389 | 0.4211 | ± | 0.7996 | 0.7464 | 0.9518 | 0.7670 | 0.0406 | ± | 0.0388 | 0.1193 | ± | 0.0632 | 0.6408 | ± | 0.2142 | 0.0066 | 0.0009 | 0.0014 |
| **N** | **Colgate Total Whole Mouth Health** | 0.0618 | ± | 0.0200 | 0.0443 | ± | 0.0186 | 0.0318 | ± | 0.0129 | 0.0445 | 0.0031 | 0.0528 | 0.5683 | ± | 0.8752 | 0.1094 | ± | 0.2025 | 0.2739 | ± | 0.4012 | 0.1412 | 0.8901 | 0.3667 | 0.0213 | ± | 0.0052 | 0.0246 | ± | 0.0249 | 0.0176 | ± | 0.0067 | 0.3456 | 0.1414 | 0.7410 |
| **O** | **Listerine Ultra Clean Zero Alcohol** | 0.0279 | ± | 0.0179 | 0.0679 | ± | 0.0457 | 0.4144 | ± | 0.0309 | 0.0113 | 0.0001 | 0.0001 | 0.0743 | ± | 0.1770 | 0.0208 | ± | 0.0456 | 0.1229 | ± | 0.3891 | 0.8354 | 0.5154 | 0.5004 | 0.0085 | ± | 0.0181 | 0.1657 | ± | 0.0833 | 0.7723 | ± | 0.0998 | 0.0002 | 0.0002 | 0.0002 |
| **P** | **Cepacol Antibacterial Multi-Protection Mouthwash** | 0.0452 | ± | 0.0190 | 0.0513 | ± | 0.0225 | 0.0526 | ± | 0.0179 | 0.4653 | 0.3025 | 0.8956 | 0.1480 | ± | 0.1814 | 0.0694 | ± | 0.1143 | 0.0068 | ± | 0.0128 | 0.4531 | 0.0428 | 0.3223 | 0.0273 | ± | 0.0177 | 0.0306 | ± | 0.0216 | 0.0383 | ± | 0.0361 | 0.8326 | 0.4476 | 0.8326 |
| **Q** | **Perio Brite Complete Oral Care Natural Mouthwash** | 0.0408 | ± | 0.0248 | 0.1519 | ± | 0.0578 | 0.2961 | ± | 0.0335 | 0.0002 | 0.0001 | 0.0002 | 0.0031 | ± | 0.0101 | 0.0133 | ± | 0.0097 | 0.0111 | ± | 0.0143 | 0.0870 | 0.1712 | 0.3140 | 0.0059 | ± | 0.0205 | 0.3043 | ± | 0.1638 | 0.7464 | ± | 0.0993 | 0.0002 | 0.0002 | 0.0002 |
| **R** | **Crest Pro-Health Intense** | 0.0000 | ± | 0.0000 | 0.0603 | ± | 0.0200 | 0.0528 | ± | 0.0121 | 0.0001 | 0.0001 | 0.2786 | 0.3662 | ± | 0.4933 | 0.4103 | ± | 0.7296 | 0.3117 | ± | 0.7017 | 0.3474 | 0.2880 | 0.4849 | 0.0367 | ± | 0.0174 | 0.0233 | ± | 0.0169 | 0.0347 | ± | 0.0537 | 0.0892 | 0.1805 | 0.7410 |
| **S** | **Hello Activated Charcoal** | 0.2532 | ± | 0.0510 | 0.4134 | ± | 0.0492 | 0.3984 | ± | 0.0332 | 0.0001 | 0.0001 | 0.6224 | 0.3400 | ± | 0.7389 | 0.0401 | ± | 0.1076 | 0.1713 | ± | 0.5456 | 0.0844 | 0.0694 | 0.5828 | 0.5332 | ± | 0.0694 | 0.7093 | ± | 0.0899 | 0.7182 | ± | 0.0565 | 0.0005 | 0.0003 | 0.7744 |
| **T** | **Jason Healthy Mouth Tartar Control Cinnamon Clove** | 0.1149 | ± | 0.1911 | 0.1226 | ± | 0.0573 | 0.2088 | ± | 0.0764 | 0.0761 | 0.0014 | 0.0274 | 0.0003 | ± | 0.0012 | 0.0093 | ± | 0.0197 | 0.1658 | ± | 0.3592 | 0.0362 | 0.0501 | 0.8186 | 0.1354 | ± | 0.1011 | 0.2185 | ± | 0.1174 | 0.3449 | ± | 0.0754 | 0.1564 | 0.0005 | 0.0174 |
| **U** | **Thera Breath Dentist Formulated Fresh Breath** | 0.0166 | ± | 0.0142 | 0.0187 | ± | 0.0187 | 0.0793 | ± | 0.0353 | 0.8796 | 0.0002 | 0.0003 | -0.0052 | ± | 0.0073 | 0.0208 | ± | 0.0660 | 0.0044 | ± | 0.0120 | 0.8040 | 0.1092 | 0.6236 | 0.0161 | ± | 0.0143 | 0.0196 | ± | 0.0256 | 0.1340 | ± | 0.0901 | 0.9699 | 0.0002 | 0.0004 |
| **V** | **Parodontax Active Gum Health Mint** | 0.0000 | ± | 0.0000 | 0.0850 | ± | 0.0105 | 0.0717 | ± | 0.0091 | 0.0001 | 0.0001 | 0.0142 | 0.3059 | ± | 0.6002 | 0.4843 | ± | 0.6468 | 0.2454 | ± | 0.5705 | 0.9518 | 0.0657 | 0.1587 | 0.0000 | ± | 0.0000 | 0.0485 | ± | 0.0234 | 0.0650 | ± | 0.0292 | 0.0002 | 0.0002 | 0.1002 |
| **W** | **Biotene Dry Mouth Oral Rinse** | 0.2088 | ± | 0.0642 | 0.3591 | ± | 0.0343 | 0.4080 | ± | 0.0433 | 0.0001 | 0.0001 | 0.0192 | 0.0181 | ± | 0.0161 | 0.1420 | ± | 0.2634 | 0.0969 | ± | 0.2675 | 0.2061 | 0.7441 | 0.5683 | 0.2517 | ± | 0.1068 | 0.5922 | ± | 0.1591 | 0.7134 | ± | 0.0696 | 0.0002 | 0.0002 | 0.0111 |
| **X** | **ARC Turn Up The Bright** | 0.0180 | ± | 0.0077 | 0.0236 | ± | 0.0094 | 0.0230 | ± | 0.0075 | 0.2034 | 0.1934 | 1.0000 | 0.3101 | ± | 0.6215 | 0.3758 | ± | 0.6631 | 0.2823 | ± | 0.5576 | 0.4631 | 0.9791 | 0.5683 | 0.0437 | ± | 0.0672 | 0.0204 | ± | 0.0101 | 0.0233 | ± | 0.0118 | 0.7928 | 0.6220 | 0.4915 |
| **Y** | **Crest Pro-Health Clean Mint Multiprotection** | 0.0033 | ± | 0.0106 | 0.0838 | ± | 0.0078 | 0.0698 | ± | 0.0133 | 0.0001 | 0.0001 | 0.0191 | 0.3713 | ± | 0.4373 | 0.2611 | ± | 0.4365 | 0.2684 | ± | 0.5389 | 0.1800 | 0.1503 | 0.7441 | 0.0120 | ± | 0.0265 | 0.0496 | ± | 0.0193 | 0.0319 | ± | 0.0066 | 0.0029 | 0.0174 | 0.0657 |
| **Z** | **Mouthwash Up and Up** | 0.0447 | ± | 0.0064 | 0.0492 | ± | 0.0047 | 0.0541 | ± | 0.0102 | 0.0596 | 0.0236 | 0.3025 | 0.4224 | ± | 0.5922 | 0.3081 | ± | 0.6137 | 0.2791 | ± | 0.5654 | 0.1800 | 0.6702 | 0.7464 | 0.0325 | ± | 0.0090 | 0.0437 | ± | 0.0144 | 0.0404 | ± | 0.0147 | 0.0735 | 0.1962 | 0.5656 |
| **AA** | **Dr. Tichenor's All Natural Peppermint Mouthwash Concentrate** | 0.0838 | ± | 0.0407 | 0.1548 | ± | 0.0362 | 0.1733 | ± | 0.0681 | 0.0015 | 0.0016 | 0.3162 | 0.0000 | ± | 0.0000 | 0.0013 | ± | 0.0021 | 0.0035 | ± | 0.0040 | 0.0715 | 0.0116 | 0.3190 | 0.1765 | ± | 0.0819 | 0.4488 | ± | 0.1072 | 0.4218 | ± | 0.1378 | 0.0002 | 0.0017 | 0.5292 |
| **AB** | **Antiseptic Mouthwash Antigingivits Antiplaque** | 0.0693 | ± | 0.0031 | 0.3385 | ± | 0.0286 | 0.4252 | ± | 0.0336 | 0.0001 | 0.0001 | 0.0002 | 0.0340 | ± | 0.0051 | 0.1136 | ± | 0.2681 | 0.0777 | ± | 0.1922 | 0.7441 | 0.0815 | 0.0870 | 0.0166 | ± | 0.0187 | 0.5812 | ± | 0.0901 | 0.8477 | ± | 0.0612 | 0.0002 | 0.0002 | 0.0002 |
| **AC** | **Hello Peace Out, Plaque** | 0.0203 | ± | 0.0063 | 0.0258 | ± | 0.0086 | 0.0325 | ± | 0.0115 | 0.1739 | 0.0113 | 0.2361 | 0.3053 | ± | 0.5749 | 0.5941 | ± | 0.8561 | 0.2721 | ± | 0.5023 | 0.5468 | 0.9438 | 0.7410 | 0.0153 | ± | 0.0095 | 0.0124 | ± | 0.0044 | 0.0131 | ± | 0.0057 | 0.7605 | 0.9153 | 0.9851 |
| **AD** | **Listerine Original** | 0.1320 | ± | 0.1817 | 0.2493 | ± | 0.0724 | 0.4313 | ± | 0.0429 | 0.0599 | 0.0041 | 0.0001 | 0.0209 | ± | 0.0118 | 0.1054 | ± | 0.2811 | 0.1043 | ± | 0.2565 | 0.3667 | 0.3014 | 0.8354 | 0.2738 | ± | 0.2739 | 0.5321 | ± | 0.2277 | 0.8036 | ± | 0.2799 | 0.0428 | 0.0042 | 0.0549 |
| **AE** | **Lavoris Freash Breath Mouthwash** | 0.3138 | ± | 0.0579 | 0.3543 | ± | 0.0593 | 0.3560 | ± | 0.0414 | 0.1524 | 0.0856 | 0.8273 | 0.0682 | ± | 0.0994 | 0.1754 | ± | 0.3027 | 0.1998 | ± | 0.5469 | 0.7726 | 0.6802 | 0.8264 | 0.6733 | ± | 0.1795 | 0.7300 | ± | 0.1632 | 0.6980 | ± | 0.1681 | 0.4621 | 0.7744 | 0.6553 |
| **AF** | **Oral B Dry mouth** | 0.2740 | ± | 0.0566 | 0.3913 | ± | 0.0248 | 0.3686 | ± | 0.0319 | 0.0002 | 0.0016 | 0.0856 | 0.3093 | ± | 0.6471 | 0.6713 | ± | 0.7098 | 0.0480 | ± | 0.0722 | 0.1800 | 0.6802 | 0.0355 | 0.3913 | ± | 0.2389 | 0.7025 | ± | 0.1896 | 0.8222 | ± | 0.3233 | 0.0153 | 0.0131 | 0.6036 |
| **AG** | **Plax Softmint Flavor Mouthwash** | 0.0580 | ± | 0.0039 | 0.0394 | ± | 0.0100 | 0.0353 | ± | 0.0078 | 0.0005 | 0.0001 | 0.5841 | 0.1863 | ± | 0.3454 | 0.3033 | ± | 0.6192 | 0.1688 | ± | 0.3285 | 0.7441 | 0.2165 | 0.1800 | 0.0004 | ± | 0.0022 | -0.0006 | ± | 0.0020 | -0.0006 | ± | 0.0028 | 0.4915 | 0.4179 | 0.8326 |
| **AH** | **ARC Fresh Breath Mouth Rinse** | 0.1317 | ± | 0.0580 | 0.1470 | ± | 0.0257 | 0.1128 | ± | 0.0282 | 0.1582 | 0.7475 | 0.0142 | 0.1529 | ± | 0.4226 | 0.1480 | ± | 0.2622 | 0.0660 | ± | 0.1096 | 0.2061 | 0.7019 | 0.3870 | 0.1533 | ± | 0.1618 | 0.1035 | ± | 0.0641 | 0.0761 | ± | 0.0333 | 0.3127 | 0.0735 | 0.4915 |
| **AI** | **Antiseptic Mouthwash Up and Up** | 0.0761 | ± | 0.0381 | 0.3122 | ± | 0.0652 | 0.3794 | ± | 0.0548 | 0.0001 | 0.0001 | 0.0341 | 0.0773 | ± | 0.1762 | 0.5054 | ± | 0.6047 | 0.3770 | ± | 0.5393 | 0.0116 | 0.0844 | 0.7441 | 0.1011 | ± | 0.1015 | 0.6718 | ± | 0.2366 | 0.8971 | ± | 0.2777 | 0.0002 | 0.0002 | 0.1002 |
| **AJ** | **Oral B Mouth Sore** | 0.0228 | ± | 0.0284 | 0.0218 | ± | 0.0110 | 0.0193 | ± | 0.0099 | 0.5011 | 0.4148 | 0.8273 | 0.4518 | ± | 0.5712 | 0.6930 | ± | 0.6238 | 0.5463 | ± | 0.7732 | 0.3140 | 0.8264 | 0.3740 | 0.0084 | ± | 0.0071 | 0.0193 | ± | 0.0125 | 0.0328 | ± | 0.0202 | 0.0231 | 0.0067 | 0.1632 |
| **AK** | **Tom`s Natural Fluoride-Free Wicked Fresh** | 0.0800 | ± | 0.0256 | 0.2603 | ± | 0.0413 | 0.3693 | ± | 0.0409 | 0.0001 | 0.0001 | 0.0003 | 0.0573 | ± | 0.1555 | 0.0129 | ± | 0.0233 | 0.0062 | ± | 0.0110 | 0.7441 | 0.7237 | 0.8901 | 0.3253 | ± | 0.1799 | 0.7179 | ± | 0.1924 | 0.9050 | ± | 0.1807 | 0.0017 | 0.0002 | 0.0403 |
| **AL** | **Listerine Cool Mint** | 0.0809 | ± | 0.0102 | 0.3182 | ± | 0.0319 | 0.3831 | ± | 0.0392 | 0.0001 | 0.0001 | 0.0015 | 0.2385 | ± | 0.5521 | 0.3577 | ± | 0.4520 | 0.3598 | ± | 0.7112 | 0.0870 | 0.7441 | 0.4849 | 0.0103 | ± | 0.0066 | 0.5799 | ± | 0.1189 | 0.9329 | ± | 0.1551 | 0.0002 | 0.0002 | 0.0005 |
| **AM** | **The Natural Dentist Healthy Gums** | 0.0528 | ± | 0.0112 | 0.1591 | ± | 0.0340 | 0.2834 | ± | 0.0320 | 0.0001 | 0.0001 | 0.0001 | 0.1407 | ± | 0.2973 | 0.4956 | ± | 0.6180 | 0.1113 | ± | 0.3465 | 0.1800 | 0.2880 | 0.0559 | 0.0600 | ± | 0.0308 | 0.3318 | ± | 0.1422 | 0.5067 | ± | 0.1802 | 0.0002 | 0.0002 | 0.0428 |
| **AN** | **Thera Breath Periodontist Formulated Healthy Gums Oral Rinse** | 0.0466 | ± | 0.0193 | 0.0488 | ± | 0.0169 | 0.0480 | ± | 0.0167 | 0.8670 | 0.8796 | 1.0000 | 0.2043 | ± | 0.3297 | 0.4126 | ± | 0.7690 | 0.5707 | ± | 0.6350 | 0.5828 | 0.3740 | 0.3667 | 0.0273 | ± | 0.0071 | 0.0353 | ± | 0.0165 | 0.0353 | ± | 0.0117 | 0.4621 | 0.1632 | 0.8198 |
| **AO** | **Oral B Dry Mouth** | 0.0470 | ± | 0.0191 | 0.0565 | ± | 0.0256 | 0.0552 | ± | 0.0261 | 0.4490 | 0.2439 | 1.0000 | 0.6339 | ± | 0.5682 | 0.0860 | ± | 0.1731 | 0.0509 | ± | 0.1103 | 0.0116 | 0.0116 | 0.3740 | 0.0150 | ± | 0.0052 | 0.0226 | ± | 0.0079 | 0.0321 | ± | 0.0171 | 0.0316 | 0.0069 | 0.1717 |
| **AP** | **Swan Mouthwash Fresh Mint** | 0.0716 | ± | 0.0242 | 0.0996 | ± | 0.1301 | 0.0598 | ± | 0.0173 | 0.7867 | 0.3554 | 0.5540 | 0.2035 | ± | 0.4993 | 0.1161 | ± | 0.2446 | 0.6363 | ± | 0.8861 | 0.9518 | 0.0524 | 0.0509 | 0.0423 | ± | 0.0165 | 0.0353 | ± | 0.0160 | 0.0503 | ± | 0.0328 | 0.3456 | 0.8038 | 0.4476 |
| **AQ** | **Crest Scope Mouthwash Rince-Bouche** | 0.0232 | ± | 0.0082 | 0.0319 | ± | 0.0089 | 0.0415 | ± | 0.0194 | 0.0468 | 0.0060 | 0.3841 | 0.2434 | ± | 0.3825 | 0.0948 | ± | 0.1310 | 0.1682 | ± | 0.4618 | 0.4792 | 0.4373 | 0.9027 | 0.0688 | ± | 0.2244 | 0.0494 | ± | 0.1098 | 0.0276 | ± | 0.0215 | 0.0549 | 0.0108 | 0.4179 |
| **AR** | **Family Wellness Blue Mint Mouthwash** | 0.0185 | ± | 0.0049 | 0.0270 | ± | 0.0079 | 0.0271 | ± | 0.0072 | 0.0179 | 0.0031 | 0.8796 | 0.3598 | ± | 0.6025 | 0.1673 | ± | 0.2880 | 0.0942 | ± | 0.1877 | 0.1800 | 0.1587 | 0.7410 | 0.0269 | ± | 0.0510 | 0.0158 | ± | 0.0062 | 0.1078 | ± | 0.1903 | 0.3456 | 0.0549 | 0.1252 |
| **AS** | **Crest All Fresh No Stress Scope All Day** | 0.1053 | ± | 0.0239 | 0.0992 | ± | 0.0182 | 0.0909 | ± | 0.0208 | 0.6036 | 0.2439 | 0.2439 | 0.0612 | ± | 0.1919 | 0.3545 | ± | 0.4625 | 0.0609 | ± | 0.1229 | 0.0220 | 0.0312 | 0.6802 | 0.1723 | ± | 0.1523 | 0.0828 | ± | 0.0298 | 0.0363 | ± | 0.0087 | 0.0029 | 0.0002 | 0.0002 |
| **AT** | **Desert Essence Tea Tree Oil Spearmint** | 0.2227 | ± | 0.0447 | 0.2794 | ± | 0.0525 | 0.2965 | ± | 0.0599 | 0.0445 | 0.0097 | 0.4000 | 0.0038 | ± | 0.0040 | 0.0145 | ± | 0.0109 | 0.1220 | ± | 0.2199 | 0.0167 | 0.0220 | 0.8264 | 0.4458 | ± | 0.0807 | 0.5611 | ± | 0.0962 | 0.5653 | ± | 0.0546 | 0.0174 | 0.0056 | 0.9153 |
| **AU** | **Jason Healthy Powersmile Brightening** | 0.0222 | ± | 0.0154 | 0.0461 | ± | 0.0178 | 0.0973 | ± | 0.0911 | 0.0121 | 0.0004 | 0.0104 | 0.0000 | ± | 0.0000 | 0.0010 | ± | 0.0016 | 0.0035 | ± | 0.0040 | 0.0715 | 0.0116 | 0.2284 | 0.0543 | ± | 0.0427 | 0.0785 | ± | 0.0680 | 0.1659 | ± | 0.1473 | 0.4915 | 0.0657 | 0.1879 |
| **AV** | **Lumineux Oral Essentials** | 0.2233 | ± | 0.0496 | 0.2710 | ± | 0.0524 | 0.2905 | ± | 0.0690 | 0.0528 | 0.0237 | 0.6825 | 0.0892 | ± | 0.3079 | 0.0028 | ± | 0.0035 | 0.0044 | ± | 0.0036 | 0.2865 | 0.0627 | 0.3740 | 0.4725 | ± | 0.0897 | 0.5611 | ± | 0.0557 | 0.4273 | ± | 0.3701 | 0.0255 | 0.6749 | 0.8038 |
| **AW** | **Jason Total Protection Sea Salt Mouthrinse** | 0.0153 | ± | 0.0188 | 0.1400 | ± | 0.0566 | 0.2090 | ± | 0.0601 | 0.0002 | 0.0001 | 0.0192 | 0.3231 | ± | 0.5333 | 0.1817 | ± | 0.2987 | 0.0204 | ± | 0.0364 | 0.7441 | 0.7721 | 0.9438 | 0.0641 | ± | 0.0309 | 0.4110 | ± | 0.1108 | 0.5998 | ± | 0.0955 | 0.0002 | 0.0002 | 0.0025 |
| **AX** | **Tea Tree Therapy Mouth Wash** | 0.1573 | ± | 0.0461 | 0.2258 | ± | 0.0363 | 0.2846 | ± | 0.0432 | 0.0034 | 0.0002 | 0.0048 | 0.2002 | ± | 0.4655 | 0.2868 | ± | 0.3157 | 0.2178 | ± | 0.4318 | 0.1529 | 0.0428 | 0.9518 | 0.4051 | ± | 0.1242 | 0.4999 | ± | 0.0809 | 0.5992 | ± | 0.0807 | 0.0549 | 0.0025 | 0.0174 |
| **AY** | **HPM Hydrogen Peroxide Mouthwash** | 0.0011 | ± | 0.0032 | 0.0044 | ± | 0.0065 | 0.0072 | ± | 0.0060 | 0.1165 | 0.0204 | 0.3376 | 0.0000 | ± | 0.0000 | 0.0000 | ± | 0.0000 | 0.0000 | ± | 0.0000 | 1.0000 | 1.0000 | 1.0000 | 0.0409 | ± | 0.0302 | 0.0343 | ± | 0.0155 | 0.0607 | ± | 0.0758 | 0.7410 | 0.8038 | 0.6411 |
| **AZ** | **Bubble Gum Kids Spry Mouthwash** | 0.0169 | ± | 0.0097 | 0.0482 | ± | 0.0280 | 0.2922 | ± | 0.0249 | 0.0048 | 0.0001 | 0.0001 | 0.0532 | ± | 0.1227 | 0.1196 | ± | 0.2992 | 0.0571 | ± | 0.1439 | 0.1006 | 0.0293 | 0.3667 | 0.0317 | ± | 0.0322 | 0.0942 | ± | 0.1263 | 0.5637 | ± | 0.0714 | 0.0107 | 0.0002 | 0.0002 |
| **BA** | **Jason Healthy Powersmile Brightening** | 0.1628 | ± | 0.0332 | 0.2466 | ± | 0.0794 | 0.2848 | ± | 0.0831 | 0.0907 | 0.0029 | 0.0599 | 0.0044 | ± | 0.0153 | 0.0119 | ± | 0.0285 | 0.0039 | ± | 0.0089 | 0.3667 | 0.3908 | 0.8594 | 0.3010 | ± | 0.1060 | 0.4898 | ± | 0.1031 | 0.6259 | ± | 0.1235 | 0.0012 | 0.0002 | 0.0174 |
| **BB** | **Swan Alcohol Free Mouthwash** | 0.2479 | ± | 0.0204 | 0.3217 | ± | 0.0223 | 0.3247 | ± | 0.0574 | 0.0001 | 0.0057 | 0.5699 | 0.0226 | ± | 0.0492 | 0.1384 | ± | 0.2969 | 0.1873 | ± | 0.3523 | 0.5468 | 0.1503 | 0.4849 | 0.5050 | ± | 0.0348 | 0.7013 | ± | 0.1043 | 0.7525 | ± | 0.2794 | 0.0002 | 0.0003 | 1.0000 |
| **BC** | **Orajel 2x Mouth Sores Rinse Medicated** | 0.0165 | ± | 0.0105 | 0.0098 | ± | 0.0083 | 0.0151 | ± | 0.0094 | 0.2108 | 0.9821 | 0.2506 | 0.3112 | ± | 0.5068 | 0.0003 | ± | 0.0009 | 0.2158 | ± | 0.3472 | 0.0428 | 0.7441 | 0.1529 | 0.0003 | ± | 0.0008 | 0.0032 | ± | 0.0056 | 0.0064 | ± | 0.0063 | 0.2335 | 0.0111 | 0.2609 |
| **BD** | **Crest Bacteria Blast** | 0.0000 | ± | 0.0000 | 0.0018 | ± | 0.0034 | 0.0055 | ± | 0.0058 | 0.1088 | 0.0056 | 0.0922 | 0.3382 | ± | 0.3992 | 0.0263 | ± | 0.0912 | 0.0393 | ± | 0.1098 | 0.0817 | 0.1457 | 0.7464 | 0.0007 | ± | 0.0013 | 0.0015 | ± | 0.0021 | 0.0027 | ± | 0.0054 | 0.4127 | 0.8114 | 0.7928 |
| **BE** | **Clean Mint Withening Alcohol Free Rinse** | 0.0001 | ± | 0.0003 | 0.0024 | ± | 0.0049 | 0.0013 | ± | 0.0023 | 0.1524 | 0.1615 | 0.9208 | 0.0000 | ± | 0.0000 | 0.0617 | ± | 0.2136 | 0.0953 | ± | 0.1893 | 0.5633 | 0.0715 | 0.2644 | 0.0247 | ± | 0.0513 | 0.0091 | ± | 0.0181 | 0.0183 | ± | 0.0084 | 0.5967 | 0.0543 | 0.0210 |
| **BF** | **Thera Breath Dentist Formulated Fresh Breath Oral Rinse** | 0.0047 | ± | 0.0061 | 0.0165 | ± | 0.0074 | 0.0731 | ± | 0.0299 | 0.0018 | 0.0001 | 0.0002 | 0.0001 | ± | 0.0003 | 0.0008 | ± | 0.0012 | 0.0096 | ± | 0.0131 | 0.1751 | 0.0011 | 0.0013 | 0.0256 | ± | 0.0204 | 0.0377 | ± | 0.0196 | 0.1383 | ± | 0.0708 | 0.1492 | 0.0002 | 0.0004 |
| **BG** | **Listerine Sensitivity Zero Alcohol** | 0.0248 | ± | 0.0065 | 0.0312 | ± | 0.0056 | 0.0339 | ± | 0.0057 | 0.0199 | 0.0028 | 0.3838 | 0.0872 | ± | 0.1838 | 0.1287 | ± | 0.2647 | 0.1656 | ± | 0.2311 | 0.7726 | 0.2375 | 0.5683 | 0.0000 | ± | 0.0000 | 0.0017 | ± | 0.0025 | 0.0105 | ± | 0.0049 | 0.0647 | 0.0002 | 0.0008 |
| **BH** | **Splendid White Whitening Mouth Rinse Alcohol Free Up and Up** | 0.0003 | ± | 0.0009 | 0.0001 | ± | 0.0003 | 0.0058 | ± | 0.0086 | 1.0000 | 0.0705 | 0.0705 | 0.3113 | ± | 0.5131 | 0.0607 | ± | 0.1435 | 0.1398 | ± | 0.4187 | 0.3605 | 0.5217 | 0.8515 | 0.0027 | ± | 0.0052 | 0.0188 | ± | 0.0344 | 0.0193 | ± | 0.0157 | 0.2280 | 0.0108 | 0.3842 |
| **BI** | **Crest Scope Classic** | 0.0323 | ± | 0.0072 | 0.0400 | ± | 0.0128 | 0.0459 | ± | 0.0155 | 0.2439 | 0.0528 | 0.3554 | 0.2273 | ± | 0.3308 | 0.0153 | ± | 0.0045 | 0.0815 | ± | 0.2419 | 0.0307 | 0.0627 | 0.3474 | 0.0138 | ± | 0.0245 | 0.0113 | ± | 0.0067 | 0.0164 | ± | 0.0106 | 0.3372 | 0.1182 | 0.1868 |
| **BJ** | **BR Rinse Organic Mouthwash** | 0.0000 | ± | 0.0000 | 0.0065 | ± | 0.0067 | 0.0070 | ± | 0.0067 | 0.0056 | 0.0023 | 0.7826 | 0.0031 | ± | 0.0107 | 0.0000 | ± | 0.0000 | 0.0002 | ± | 0.0004 | 0.5633 | 0.7950 | 0.3474 | 0.0286 | ± | 0.0129 | 0.0388 | ± | 0.0129 | 0.0806 | ± | 0.0892 | 0.1189 | 0.0069 | 0.3127 |
| **BK** | **Tom's Sea Salt** | 0.0213 | ± | 0.0117 | 0.2081 | ± | 0.0518 | 0.2639 | ± | 0.0849 | 0.0001 | 0.0006 | 0.0045 | 0.0007 | ± | 0.0010 | 0.0161 | ± | 0.0071 | 0.0169 | ± | 0.0084 | 0.0013 | 0.0013 | 0.6802 | 0.1266 | ± | 0.0433 | 0.5575 | ± | 0.1905 | 0.5887 | ± | 0.1493 | 0.0002 | 0.0002 | 0.1717 |
| **BL** | **CloSYS** | 0.0092 | ± | 0.0102 | 0.0433 | ± | 0.0177 | 0.1436 | ± | 0.0319 | 0.0006 | 0.0001 | 0.0001 | 0.0000 | ± | 0.0000 | 0.0000 | ± | 0.0000 | 0.0070 | ± | 0.0020 | 1.0000 | 0.0011 | 0.0011 | 0.0543 | ± | 0.0108 | 0.0793 | ± | 0.0149 | 0.4003 | ± | 0.1001 | 0.0020 | 0.0002 | 0.0002 |
| **BM** | **Antiseptic Mouthwash Antigingivits Antiplaque** | 0.0138 | ± | 0.0109 | 0.2630 | ± | 0.0258 | 0.4370 | ± | 0.0318 | 0.0001 | 0.0001 | 0.0001 | 0.0042 | ± | 0.0057 | 0.0166 | ± | 0.0051 | 0.0191 | ± | 0.0126 | 0.0091 | 0.0081 | 0.8201 | 0.0018 | ± | 0.0034 | 0.2778 | ± | 0.1280 | 0.4259 | ± | 0.1011 | 0.0002 | 0.0002 | 0.0069 |
| **BN** | **Crest Gum and Breath Purify** | 0.0990 | ± | 0.0509 | 0.1945 | ± | 0.0341 | 0.1546 | ± | 0.0181 | 0.0012 | 0.0041 | 0.0020 | 0.2068 | ± | 0.5178 | 0.0471 | ± | 0.1178 | 0.1638 | ± | 0.2554 | 0.7441 | 0.0657 | 0.0249 | 0.0142 | ± | 0.0264 | 0.1291 | ± | 0.0189 | 0.0907 | ± | 0.0195 | 0.0002 | 0.0002 | 0.0018 |
| **BO** | **Swan Antiseptic Mouth Rinse Original** | 0.1229 | ± | 0.1514 | 0.2334 | ± | 0.0333 | 0.3453 | ± | 0.0624 | 0.0636 | 0.0068 | 0.0001 | 0.0094 | ± | 0.0063 | 0.0193 | ± | 0.0037 | 0.0118 | ± | 0.0032 | 0.0119 | 0.4849 | 0.0078 | 0.0619 | ± | 0.1251 | 0.2994 | ± | 0.1266 | 0.5092 | ± | 0.1487 | 0.0029 | 0.0002 | 0.0012 |
| **BP** | **Swan Antiseptic Mouth Rinse Spring Mint** | 0.0295 | ± | 0.0105 | 0.2628 | ± | 0.0318 | 0.3558 | ± | 0.0456 | 0.0001 | 0.0001 | 0.0005 | 0.0129 | ± | 0.0042 | 0.1803 | ± | 0.4167 | 0.0149 | ± | 0.0028 | 0.0092 | 0.4598 | 0.0140 | 0.0338 | ± | 0.0518 | 0.3733 | ± | 0.0807 | 0.4962 | ± | 0.0808 | 0.0002 | 0.0002 | 0.0069 |
| **BQ** | **Advanced Mouth Rinse** | 0.0531 | ± | 0.0107 | 0.1867 | ± | 0.0316 | 0.3637 | ± | 0.0386 | 0.0001 | 0.0001 | 0.0001 | 0.1234 | ± | 0.3353 | 0.0253 | ± | 0.0087 | 0.0248 | ± | 0.0083 | 0.1213 | 0.1712 | 0.7904 | 0.0013 | ± | 0.0021 | 0.3102 | ± | 0.0843 | 0.6015 | ± | 0.1600 | 0.0002 | 0.0002 | 0.0002 |
| **BR** | **Crest 3D White Brilliance** | 0.0095 | ± | 0.0068 | 0.0188 | ± | 0.0110 | 0.0281 | ± | 0.0111 | 0.0445 | 0.0015 | 0.0761 | 0.0003 | ± | 0.0006 | 0.1348 | ± | 0.3502 | 0.1416 | ± | 0.3431 | 0.4598 | 0.1800 | 0.7464 | 0.0037 | ± | 0.0059 | 0.0008 | ± | 0.0012 | 0.0027 | ± | 0.0053 | 0.3127 | 0.7102 | 0.6412 |
| **BS** | **Multi-Action Alcohol Free Antiseptic Mouth Rinse** | 0.0128 | ± | 0.0281 | 0.1434 | ± | 0.0174 | 0.1089 | ± | 0.0166 | 0.0001 | 0.0001 | 0.0005 | 0.0735 | ± | 0.0680 | 0.1471 | ± | 0.2430 | 0.0183 | ± | 0.0278 | 0.7464 | 0.0116 | 0.0116 | 0.0000 | ± | 0.0000 | 0.0527 | ± | 0.0240 | 0.0332 | ± | 0.0084 | 0.0002 | 0.0002 | 0.0579 |
| **BT** | **BR Rinse Organic Mouthwash** | 0.0143 | ± | 0.0158 | 0.0145 | ± | 0.0151 | 0.0128 | ± | 0.0176 | 0.8956 | 0.8580 | 0.5544 | 0.0000 | ± | 0.0000 | 0.0000 | ± | 0.0000 | 0.0000 | ± | 0.0000 | 1.0000 | 1.0000 | 1.0000 | 0.0250 | ± | 0.0120 | 0.1058 | ± | 0.2472 | 0.1456 | ± | 0.2747 | 0.0735 | 0.2738 | 0.5656 |
| **BU** | **Guru Nanda Oxyburst Whitening Technology** | 0.0065 | ± | 0.0087 | 0.0083 | ± | 0.0118 | 0.0158 | ± | 0.0152 | 1.0000 | 0.1557 | 0.1582 | 0.0000 | ± | 0.0000 | 0.0000 | ± | 0.0000 | 0.0000 | ± | 0.0000 | 1.0000 | 1.0000 | 1.0000 | 0.0312 | ± | 0.0512 | 0.0138 | ± | 0.0087 | 0.0195 | ± | 0.0114 | 0.3127 | 0.9699 | 0.3456 |
| **BV** | **PerCara Mouthwash and Gargle Refresh Mint** | 0.0319 | ± | 0.0162 | 0.0427 | ± | 0.0164 | 0.0458 | ± | 0.0126 | 0.1138 | 0.0237 | 0.5540 | 0.0353 | ± | 0.0643 | 0.1578 | ± | 0.2609 | 0.1478 | ± | 0.4424 | 0.5631 | 0.1457 | 0.1712 | 0.0337 | ± | 0.0140 | 0.0311 | ± | 0.0307 | 0.0302 | ± | 0.0083 | 0.2629 | 0.9699 | 0.2390 |
| **BW** | **Spry Oral Rinse (Dental Defense System)** | 0.2678 | ± | 0.0357 | 0.3589 | ± | 0.0164 | 0.3434 | ± | 0.0201 | 0.0001 | 0.0003 | 0.0808 | 0.0253 | ± | 0.0330 | 0.0112 | ± | 0.0134 | 0.0118 | ± | 0.0117 | 0.0524 | 0.1587 | 0.8354 | 0.2572 | ± | 0.1076 | 0.4943 | ± | 0.1176 | 0.5002 | ± | 0.1082 | 0.0009 | 0.0008 | 0.9851 |
| **BX** | **Desert Essence Tea Tree Oil Whitening Plus Mouthwash** | 0.2611 | ± | 0.0233 | 0.3649 | ± | 0.0274 | 0.4193 | ± | 0.0239 | 0.0001 | 0.0001 | 0.0012 | 0.1318 | ± | 0.4523 | 0.0408 | ± | 0.0880 | 0.1211 | ± | 0.2032 | 0.0211 | 0.0293 | 0.9791 | 0.2937 | ± | 0.1216 | 0.5083 | ± | 0.1251 | 0.4281 | ± | 0.0688 | 0.0029 | 0.0111 | 0.1002 |
| **BY** | **Desert Essence Prebiotic Plant Based Brushing Rinse Mint** | 0.2364 | ± | 0.0128 | 0.3671 | ± | 0.0160 | 0.4167 | ± | 0.0251 | 0.0001 | 0.0001 | 0.0003 | 0.1438 | ± | 0.4766 | 0.0144 | ± | 0.0021 | 0.0111 | ± | 0.0081 | 0.0965 | 0.5468 | 0.0524 | 0.3218 | ± | 0.0858 | 0.4506 | ± | 0.0676 | 0.5852 | ± | 0.0598 | 0.0029 | 0.0002 | 0.0007 |
| **BZ** | **Smart Mouth Sore Zinc Activatted Oral Rinse** | 0.0061 | ± | 0.0066 | 0.0132 | ± | 0.0086 | 0.0484 | ± | 0.0140 | 0.0991 | 0.0001 | 0.0002 | 0.0005 | ± | 0.0012 | 0.0152 | ± | 0.0354 | 0.0045 | ± | 0.0131 | 0.5468 | 0.4373 | 0.9791 | 0.0000 | ± | 0.0000 | 0.0000 | ± | 0.0000 | 0.0846 | ± | 0.0185 | 1.0000 | 0.0002 | 0.0002 |
| **CA** | **Smart Mouth Original** | 0.0021 | ± | 0.0033 | 0.0158 | ± | 0.0128 | 0.0629 | ± | 0.0138 | 0.0006 | 0.0001 | 0.0002 | 0.0005 | ± | 0.0017 | 0.0101 | ± | 0.0340 | 0.3124 | ± | 0.7273 | 0.7464 | 0.0116 | 0.0293 | 0.0027 | ± | 0.0092 | 0.0175 | ± | 0.0267 | 0.1188 | ± | 0.0317 | 0.0106 | 0.0002 | 0.0003 |
| **CB** | **Smart Mouth Clinical Zinc Activated Oral Rinse** | 0.0078 | ± | 0.0088 | 0.0303 | ± | 0.0157 | 0.0240 | ± | 0.0103 | 0.0014 | 0.0015 | 0.3291 | 0.6565 | ± | 0.8365 | 0.0554 | ± | 0.1600 | 0.4536 | ± | 0.7321 | 0.9791 | 0.8869 | 0.7441 | 0.0002 | ± | 0.0006 | 0.1144 | ± | 0.2429 | 0.0222 | ± | 0.0128 | 0.0003 | 0.0002 | 0.3752 |
| **CC** | **Parodontax Active Gum Health Clear Mint** | 0.0109 | ± | 0.0210 | 0.1335 | ± | 0.0166 | 0.1028 | ± | 0.0168 | 0.0001 | 0.0001 | 0.0015 | 0.2783 | ± | 0.3606 | 0.1373 | ± | 0.4276 | 0.2369 | ± | 0.4129 | 0.0116 | 0.1977 | 0.9027 | 0.0000 | ± | 0.0000 | 0.0701 | ± | 0.0254 | 0.0558 | ± | 0.0129 | 0.0002 | 0.0002 | 0.3372 |
