# Supplementary material for: In vitro effect of fluoride-free mouthwashes on Streptococcus mutans biofilm
Source: Clin Oral Investig. 2025 Aug 14;29(9):413. doi: 10.1007/s00784-025-06462-7 (PMC12354505; doi:10.1007/s00784-025-06462-7)
Supplement: Supplementary file 3 — Supplementary file3 (DOCX 28 KB) [file 784_2025_6462_MOESM3_ESM.docx]

**Appendix 3:** MBC and percentage of reduction of values compared with the negative control, and the statistical difference in the first dilution (1:3) of each mouthwash with other mouthwashes for total absorbance and biofilm analysis.

| **Code** | **MBC** |  | **Total absorbance analyses 1:3 dilution** | |  | **Biofilm analysis 1:3 dilution** | |
| --- | --- | --- | --- | --- | --- | --- | --- |
|  |  |  | **% of reduction comparative with negative control** | **Statistical difference p value < 0.05; (code of the other mouthwash)** |  | **% of reduction comparative with negative control** | **Statistical differences p value < 0.05; (code of the other mouthwash)** |
| **A** | >1:12 |  | 90.20 | B, C, D, E, F, G, H, I, K, L, M, N, O, R, S, T, U, V, W, X, Y, AA, AB, AC, AE, AF, AH, AI, AJ, AK, AL, AP, AQ, AR, AS, AT, AU, AV, AW, AX, AY, AZ, BA, BB, BC, BD, BE, BF, BG, BH, BI, BJ, BK, BL, BM, BN, BP, BR, BS, BT, BU, BV, BW, BX, BY, BZ, CA, CB, CC |  | 98.42 | B, D, E, F G, J, L, M, N, Q, R, S, T, V, W, Z, AA, AD, AE, AF, AG, AH, AI, AK, AM, AN, AP, AS, AT, AU, AV, AW, AX, AY, AZ, BA, BB, BC, BD, BG, BH, BJ, BK, BL, BM, BQ, BR, BS, BT, BV, BW, BX, BY, BZ, CA, CB, CC |
| **B** | <1:3 |  | 40.56 | A, C, D, E, F, G, H, I, J, K, L, M, N, O, P, Q, R, T, U, V, W, X, Y, Z, AA, AB, AC, AD, AG, AH, AI, AJ, AK, AL, AM, AN, AO, AP, AQ, AR, AS, AT, AU, AV, AW, AX, AY, AZ, BA, BB, BC, BD, BE, BF, BG, BH, BI, BJ, BK, BL, BM, BN, BO, BP, BQ, BR, BS, BT, BU, BV, BY, BZ, CA, CB, CC |  | 42.34 | A, C, D, E, F, G, H, I, J, K, M, N, O, P, Q, R, T, U, V, W, X, Y, Z, AA, AB, AC, AE, AG, AH, AI, AJ, AL, AM, AN, AO, AP, AQ, AR, AS, AU, AW, AY AZ, BA, BC, BD, BE, BF, BG, BH, BI, BJ, BK, BL, BM, BN, BO, BP, BQ, BR, BS, BT, BU, BV, BW, BX, BY, BZ, CA, CB, CC |
| **C** | >1:12 |  | 99.06 | A, B, D, I, J, L, M, N, O, P, Q, S, T, U, W, X, Z, AA, AB, AC, AD, AE, AF, AG, AH, AI, AJ, AK, AL, AM, AN, AO, AP, AQ, AR, AS, AT, AU, AV, AX, AZ, BA, BB, BC, BG, BI, BK, BM, BN, BO, BP, BQ, BV, BW, BX, BY |  | 93.80 | B, D, E, F, G, L, Q, R, S, T, V, W, Z, AA, AD, AE, AF, AG, AH, AI, AK, AM, AP, AS, AT AU, AV, AW, AX, AY, BA, BB, BC, BD, BG, BH, BK, BL, BM, BQ, BS, BW, BX, BY, BZ, CA, CB, CC |
| **D** | >1:12 |  | 95.77 | A, B, C, E, F, G, H, I, J, L, N, P, Q, R, S, T, V, W, Y, Z, AA, AB, AD, AE, AF, AG, AH, AI, AK, AL, AM, AN, AO, AP, AS, AT, AV, AX, AY, BA, BB, BD, BE, BF, BH, BI, BJ, BL, BN, BP, BQ, BR, BS, BU, BV, BW, BX, BY, BZ, CA, CB |  | 99.98 | A, B, C, H, I, K, L, M, N, P, R, S, T, U, W, X, Y, Z, AA, AB, AC, AD, AE, AF, AH, AI, AJ ,AK, AL, AM, AN, AO, AP, AR, AS, AT AU, AV, AW, AX, AY AZ, BA, BB, BF, BI, BJ, BK, BL, BM, BP, BT, BU, BV, BW, BX, BY |
| **E** | >1:12 |  | 100.00 | A, B, D, H, I, J, K, L, M, N, O, P, Q, S, T, U, W, X, Z, AA, AB, AC, AD, AE, AF, AG, AH, AI, AJ, AK, AL, AM, AN, AO, AP, AQ, AR, AS, AT, AU, AV, AW, AX, AZ, BA, BB, BC, BF, BG, BI, BK, BL, BM, BN, BO, BP, BQ, BR, BT, BU, BV, BW, BX, BY, BZ, CB, CC |  | 100.00 | A, B, C, H, I, K, L, M, N, P, R, S, T, U, W, X, Y, Z, AA, AB, AC, AD, AE, AF, AH, AI, AJ ,AK, AL, AM, AN, AO, AP, AQ, AR, AS, AT AU, AV, AW, AX, AY, AZ, BA, BB, BE, BF, BI, BJ, BK, BL, BM, BO, BP, BR, BT, BU, BV, BW, BX, BY |
| **F** | >1:12 |  | 99.11 | A, B, D, H, I, J, K, L, M, N, O, P, Q, S, T, U, W, X, Z, AA, AB, AC, AD, AE, AF, AG, AH, AI, AJ, AK, AL, AM, AN, AO, AP, AQ, AR, AS, AT, AU, AV, AX, AZ, BA, BB, BC, BG, BI, BK, BM, BN, BO, BP, BQ, BR, BV, BW, BX, BY |  | 100.00 | A, B, C, H, I, K, L, M, N, P, R, S, T, U, W, X, Y, Z, AA, AB, AC, AD, AE, AF, AH, AI, AJ ,AK, AL, AM, AN, AO, AP, AQ, AR, AS, AT, AU, AV, AW, AX, AY, AZ, BA, BB, BE, BF, BI, BJ, BK, BL, BO, BP, BR, BT, BU, BV, BX, BW, BY |
| **G** | >1:12 |  | 100.00 | A, B, D, H, I, J, K, L, M, N, O, P, Q, S, T, U, W, X, Z, AA, AB, AC, AD, AE, AF, AG, AH, AI, AJ, AK, AL, AM, AN, AO, AP, AQ, AR, AS, AT, AU, AV, AW, AX, AZ, BA, BB, BC, BF, BG, BI, BK, BL, BM, BN, BO, BP, BQ, BR, BT, BU, BV, BW, BX, BY, BZ, CB, CC |  | 100.00 | A, B, C, H, I, K, L, M, N, P, R, S, T, U, W, X, Y, Z, AA, AB, AC, AD, AE, AF, AH, AI, AJ ,AK, AL, AM, AN, AO, AP, AQ, AR, AS, AT AU, AV, AW, AX, AY AZ, BA, BB, BE, BF, BI, BJ, BK, BL, BO, BP, BR, BT, BU, BV, BW, BX, BY |
| **H** | <1:3 |  | 98.54 | A, B, D, E, G, I, J, L, M, N, O, P, Q, R, S, T, V, W, X, Y, Z, AA, AB, AC, AD, AE, AF, AG, AH, AI, AJ, AK, AL, AM, AN, AO, AP, AQ, AR, AS, AT, AU, AV, AX, AY, AZ, BA, BB, BC, BD, BE, BG, BH, BI, BJ, BK, BN, BO, BP, BQ, BV, BW, BX |  | 97.45 | B, D, E, F, G, L, M, R, S, T, V, W, Z, AA, AD, AE, AF, AG, AH, AI, AK, AM, AN,AP, AS, AT AU, AV, AW, AX, AY, BA, BB, BC, BD,BG, BJ, BK, BL, BQ, BS, BV, BW, BX, BY, BZ, CB, CC |
| **I** | <1:3 |  | 93.07 | A, B, C, D, E, F, G, H, J, K, L, N, R, S, T, U, V, W, X, Y, Z, AA, AB, AC, AE, AF, AG, AH, AI, AJ, AK, AL, AM, AN, AP, AQ, AR, AS, AT, AV, AW, AX, AY, AZ, BA, BB, BC, BD, BE, BF, BG, BH, BJ, BK, BL, BM, BN, BQ, BR, BS, BT, BU, BW, BX, BY, BZ, CA, CB, CC |  | 83.33 | B, D, E, F, G, I, J, L, M, Q, S, T, V, W, Z, AA, AD, AE, AF, AG, AH, AI, AK, AM, AN, AP, AS, AT AU, AV, AW, AX, AY, BA, BB, BC, BD, BG, BH, BK, BL, BM, BQ, BR, BS, BV, BW, BX, BY, BZ, CA, CB, CC |
| **J** | <1:6 |  | 90.08 | B, C, D, E, F, G, H, I, K, L, M, N, O, R, S, T, U, V, W, X, Y, AA, AB, AC, AE, AF, AG, AH, AI, AJ, AK, AL, AM, AP, AQ, AR, AS, AT, AU, AV, AW, AX, AY, AZ, BA, BB, BC, BD, BE, BF, BG, BH, BI, BJ, BK, BL, BM, BN, BP, BR, BS, BT, BU, BV, BW, BX, BY, BZ, CA, CB, CC |  | 99.58 | A, B, I, K, L, M, N, P, R, S, T, U, W, X, Z, AA, AB, AC, AD, AE, AF, AH, AI, AK, AL, AM, AN, AO, AP, AR, AS, AT AU, AV, AW, AX, AY AZ, BA, BB, BF, BJ, BK, BL, BP, BT, BU, BV, BW, BX, BY |
| **K** | >1:12 |  | 97.29 | A, B, E, F, G, I, J, L, M, N, P, Q, R, S, T, V, W, Y, Z, AA, AB, AC, AD, AE, AF, AG, AH, AI, AK, AL, AM, AN, AO, AP, AQ, AS, AT, AU, AV, AX, AY, BA, BB, BD, BE, BG, BH, BI, BJ, BN, BO, BP, BQ, BS, BV, BW, BX, BY, CA |  | 98.81 | B, D, E, F, G, J, L, M, N, P, Q, R, S, T, V, W, Z, AA, AD, AE, AF, AG, AH, AI, ,AK, AL, AM, AN, AO, AP, AS, AT AU, AV, AW, AX, AY AZ, BA, BB, BC, BD, BF, BG, BH, BJ, BK, BL, BM, BQ, BR, BS, BT, BV, BW, BX, BY, BZ, CA, CB, CC |
| **L** | <1:6 |  | 65.78 | A, B, C, D, E, F, G, H, I, J, K, M, N, O, P, Q, R, S, T, U, V, W, X, Y, Z, AA, AB, AC, AE, AF, AG, AH, AI, AJ, AK, AL, AM, AN, AO, AP, AQ, AR, AS, AT, AU, AV, AW, AY, AZ, BB, BC, BD, BE, BF, BG, BH, BI, BJ, BK, BL, BM, BN, BP, BQ, BR, BS, BT, BU, BV, BW, BX, BY, BZ, CA, CB, CC |  | 49.19 | A, C, D, E, F, G, H, I, J, K, M, N, O, P, Q, R, S, T, U, V, W, X, Y, Z, AA, AB, AC, AE, AG, AH, AI, AJ, AL, AM, AN, AO, AP, AQ, AR, AS, AU, AW, AY, AZ, BB, BC, BD, BE, BF, BG, BH, BI, BJ, BK, BL, BM, BN, BO, BP, BQ, BR, BS, BT, BU, BV, BW, BZ, CA, CB, CC |
| **M** | <1:6 |  | 94.60 | A, B, C, E, F, G, H, J, K, L, N, P, R, S, T, V, W, Y, Z, AA, AB, AE, AF, AG, AH, AI, AK, AL, AM, AN, AO, AP, AS, AT, AV, AX, AY, BA, BB, BD, BE, BF, BH, BJ, BL, BN, BQ, BR, BS, BU, BW, BX, BY, BZ, CA, CB, CC |  | 94.76 | A, B, C, D, E, F, G, H, I, J, K, L, N, O, Q, S, U, V, W, Y, AA, AB, AC, AD, AE, AF, AG, AH, AJ ,AK, AL, AO, AP, AQ, AR, AS, AT, AV, AW, AX, BA, BB, BC, BD, BE, BG, BH, BI, BK, BL, BM, BN, BO, BQ, BR, BS, BU, BW, BX, BY, BZ, CA, CB, CC |
| **N** | >1:12 |  | 86.59 | A, B, C, D, E, F, G, H, I, J, K, L, M, O, Q, R, S, U, V, W, X, Y, Z, AC, AE, AF, AH, AJ, AL, AQ, AR, AS, AT, AU, AV, AW, AX, AY, AZ, BA, BB, BC, BD, BE, BF, BG, BH, BI, BJ, BK, BL, BM, BN, BP, BR, BS, BT, BU, BV, BW, BX, BY, BZ, CA, CB, CC |  | 97.26 | A, B, D, E, F, G, J, K, L, M, N, Q, S, T, V, W, Z, AA, AB, AD, AE, AF, AG, AH, AI, AJ ,AK, AL, AM, AO, AP, AQ, AR, AS, AT, AU, AV, AW, AX, AY, BA, BB, BC, BD, BE, BG, BH, BI, BK, BL, BM, BN, BQ, BR, BS, BV, BW, BX, BY, BZ, CA, CB, CC |
| **O** | >1:3 |  | 93.95 | A, B, C, E, F, G, H, J, L, N, P, R, S, T, V, W, Y, Z, AA, AB, AE, AF, AG, AH, AI, AK, AL, AM, AO, AP, AS, AT, AV, AX, AY, BA, BB, BD, BE, BF, BH, BJ, BL, BN, BQ, BR, BS, BT, BU, BW, BX, BY, BZ, CA, CB |  | 98.90 | B, L, M, P, R, S, T, W, Z, AA, AD, AE, AF,AH, AI, AK, AM, AN, AP, AS, AT, AU, AV, AW, AX, AY, AZ, BA, BB, BJ, BK, BL, BT, BV, BW, BX, BY |
| **P** | >1:12 |  | 90.20 | B, C, D, E, F, G, H, K, L, M, O, R, S, U, V, W, X, Y, AA, AB, AC, AE, AF, AH, AI, AJ, AK, AL, AP, AQ, AR, AS, AT, AU, AV, AW, AX, AY, AZ, BA, BB, BC, BD, BE, BF, BG, BH, BJ, BK, BL, BM, BN, BR, BS, BT, BU, BW, BX, BY, BZ, CA, CB, CC |  | 96.48 | B, D, E, F, G, J, K, L, O, Q, S, T, V, W, AA, AD, AE, AF, AG, AH, AI, AJ ,AK, AM, AQ, AS, AT, AV, AW, AX, BA, BB, BC, BD, BE, BG, BH, BK, BL, BM, BN, BQ, BR, BS, BW, BX, BY, BZ, CA, CB, CC |
| **Q** | <1:3 |  | 91.16 | B, C, D, E, F, G, H, K, L, N, R, S, T, U, V, W, X, Y, AA, AB, AC, AE, AF, AG, AH, AI, AJ, AK, AL, AP, AR, AS, AT, AV, AW, AX, AY, AZ, BA, BB, BC, BD, BE, BF, BH, BJ, BK, BL, BM, BN, BP, BR, BS, BT, BU, BW, BX, BY, BZ, CA, CB, CC |  | 99.23 | A, B, C, I, L, M, N, P, R, S, T, U, W, X, Z, AA, AB, AC, AD, AE, AF, AH, AI, AJ ,AK, AL, AM, AN, AO, AP, AR, AS, AT, AU, AV, AW, AX, AY, AZ, BA, BB, BF, BI, BJ, BK, BL, BO, BP, BR, BT, BU, BV, BW, BX, BY |
| **R** | >1:12 |  | 100.00 | A, B, D, H, I, J, K, L, M, N, O, P, Q, S, T, U, W, X, Z, AA, AB, AC, AD, AE, AF, AG, AH, AI, AJ, AK, AL, AM, AN, AO, AP, AQ, AR, AS, AT, AU, AV, AW, AX, AZ, BA, BB, BC, BF, BG, BI, BK, BL, BM, BN, BO, BP, BQ, BR, BT, BU, BV, BW, BX, BY, BZ, CB, CC |  | 95.27 | A, B, C, D, E, F, G, H, J, K, L, O, Q, S, T, U, V, W, Y, AA, AB, AC, AD, AE, AF, AG, AH, AJ ,AK, AL, AO, AQ, AR, AS, AT, AV, AX, BA, BB, BC, BD, BE, BG, BH, BI, BK, BL, BM, BN, BQ, BR, BS, BW, BX, BY, BZ, CA, CB, CC |
| **S** | <1:3 |  | 45.12 | A, C, D, E, F, G, H, I, J, K, L, M, N, O, P, Q, R, T, U, V, X, Y, Z, AA, AB, AC, AD, AE, AG, AH, AI, AJ, AK, AL, AM, AN, AO, AP, AQ, AR, AS, AU, AW, AX, AY, AZ, BA, BC, BD, BE, BF, BG, BH, BI, BJ, BK, BL, BM, BN, BO, BP, BQ, BR, BS, BT, BU, BV, BZ, CA, CB, CC |  | 31.23 | A, C, D, E, F, G, H, I, J, K, L, M, N, O, P, Q, R, T, U, V, W, X, Y, Z, AA, AB, AC, AD, AE, AG, AH, AI, AJ ,AK, AL, AM, AN, AO, AP, AQ, AR, AS, AT, AU, AW, AX, AY, AZ, BA, BC, BD, BE, BF, BG, BH, BI, BJ, BK, BL, BM, BN, BO, BP, BQ, BR, BS, BT, BU, BV, BX, BW, BY, BZ, CA, CB, CC |
| **T** | <1:3 |  | 75.07 | A, B, C, D, E, F, G, H, I, J, K, L, M, O, Q, R, S, U, V, W, X, Y, Z, AC, AE, AF, AH, AJ, AL, AN, AQ, AR, AS, AT, AU, AV, AW, AX, AY, AZ, BA, BB, BC, BD, BE, BF, BG, BH, BI, BJ, BK, BL, BM, BP, BR, BS, BT, BU, BV, BW, BX, BY, BZ, CA, CB, CC |  | 82.53 | A, B, C, D, E, F, G, H, I, J, K, L, N, O, P, Q, R, S, T, V, W, X, Y, AB, AC, AE, AF, AG, AJ ,AK, AL, AN, AO, AQ, AR, AT, AV, AX, AZ, BA, BB, BC, BD, BE, BF, BG, BH, BI, BJ, BM, BN, BO, BP, BQ, BR, BS, BT, BU, BW, BX, BY, BZ, CA, CB, CC |
| **U** | >1:12 |  | 96.40 | A, B, C, E, F, G, I, J, L, N, P, Q, R, S, T, V, W, Y, Z, AA, AB, AD, AE, AF, AG, AH, AI, AK, AL, AM, AN, AO, AP, AS, AT, AV, AX, AY, BA, BB, BD, BE, BH, BI, BJ, BN, BP, BQ, BV, BW, BX, BY, CA |  | 97.92 | B, D, E, F, G, J, L, M, Q, R, S, T, V, W, Z, AA, AD, AE, AF, AG, AH, AI, ,AK, AM, AP, AS, AT, AU, AV, AW, AX, AY, BA, BB, BC, BD, BG, BH, BK, BL, BQ, BR, BS, BT, BU, BV, BW, BX, BY, BZ, CA, CB, CC |
| **V** | >1:12 |  | 100.00 | A, B, D, H, I, J, K, L, M, N, O, P, Q, S, T, U, W, X, Z, AA, AB, AC, AD, AE, AF, AG, AH, AI, AJ, AK, AL, AM, AN, AO, AP, AQ, AR, AS, AT, AU, AV, AW, AX, AZ, BA, BB, BC, BF, BG, BI, BK, BL, BM, BN, BO, BP, BQ, BR, BT, BU, BV, BW, BX, BY, BZ, CB, CC |  | 100.00 | A, B, C, H, I, K, L, M, N, P, R, S, T, U, W, X, Y, Z, AA, AB, AC, AD, AE, AF, AH, AI, AJ ,AK, AL, AM, AN, AO, AP, AQ, AR, AS, AT, AU, AV, AW, AX, AY, AZ, BA, BB, BE, BF, BI, BJ, BK, BL, BM, BO, BP, BR, BT, BU, BV, BW, BX, BY |
| **W** | <1:3 |  | 54.70 | A, B, C, D, E, F, G, H, I, J, K, L, M, N, O, P, Q, R, T, U, V, X, Y, Z, AA, AB, AC, AE, AF, AG, AH, AI, AJ, AK, AL, AM, AN, AO, AP, AQ, AR, AS, AU, AW, AX, AY, AZ, BA, BC, BD, BE, BF, BG, BH, BI, BJ, BK, BL, BM, BN, BP, BQ, BR, BS, BT, BU, BV, BW, BX, BZ, CA, CB, CC |  | 67.53 | A, B, C, D, E, F, G, H, I, J, K, L, M, N, O, P, Q, R, S, T, U, V, X, Y, Z, AB, AC, AE, AG, AH, AI, AJ, AL, AM, AN, AO, AP, AQ, AR, AS, AT, AU, AV, AW, AX, AY, AZ, BB, BC, BD, BE, BF, BG, BH, BI, BJ, BK, BL, BM, BN, BO, BP, BQ, BR, BS, BT, BU, BV, BZ, CA, CB, CC |
| **X** | >1:12 |  | 96.10 | A, B, C, E, F, G, H, I, J, L, N, P, Q, R, S, T, V, W, Y, Z, AA, AB, AD, AE, AF, AG, AH, AI, AK, AL, AM, AN, AO, AP, AS, AT, AV, AX, AY, BA, BB, BD, BE, BF, BH, BI, BJ, BL, BN, BP, BQ, BR, BS, BU, BV, BW, BX, BY, BZ, CA, CB |  | 94.37 | B, D, E, F, G, J, L, Q, S, T, V, W, AA, AD, AE, AF, AG, AH, AI, AK, AP, AQ, AS, AT, AU, AV, AW, AX, BA, BB, BC, BD, BG, BK, BL, BS, BV, BW, BX, BY, BZ, CB, CC |
| **Y** | >1:12 |  | 99.35 | A, B, D, H, I, J, K, L, M, N, O, P, Q, S, T, U, W, X, Z, AA, AB, AC, AD, AE, AF, AG, AH, AI, AJ, AK, AL, AM, AN, AO, AP, AQ, AR, AS, AT, AU, AV, AW, AX, AZ, BA, BB, BC, BG, BI, BK, BL, BM, BN, BO, BP, BQ, BR, BV, BW, BX, BY, BZ, CB |  | 98.45 | B, D, E, F, G, L, M, R, S, T, V, W, Z, AA, AB, AC, AD, AE, AF, AG, AH, AI, AJ ,AK, AL, AM, AN, AO, AP, AQ, AR, AS, AT, AU, AV, AW, AX, AY, AZ, BA, BB, BC, BD, BE, BF, BG, BH, BI, BJ, BK, BL, BM, BN, BO, BP, BQ, BR, BS, BT, BU, BV, BW, BX, BY, BZ, CA, CB, CC |
| **Z** | >1:12 |  | 90.31 | B, C, D, E, F, G, H, I, K, L, M, N, O, R, S, T, U, V, W, X, Y, AA, AB, AC, AE, AF, AG, AH, AI, AJ, AK, AL, AM, AP, AQ, AR, AS, AT, AU, AV, AW, AX, AY, AZ, BA, BB, BC, BD, BE, BF, BG, BH, BI, BJ, BK, BL, BM, BN, BP, BR, BS, BT, BU, BV, BW, BX, BY, BZ, CA, CB, CC |  | 95.81 | A, B, C, D, E, F, G, H, I, J, K, L, N, O, Q, S, U, V, W, Y, AA, AB, AC, AD, AE, AF, AG, AH, AI, AJ ,AK, AL, AO, AQ, AR, AS, AT, AV, AW, AX, BA, BB, BC, BD, BE, BG, BH, BI, BK, BL, BM, BN, BQ, BR, BS, BU, BW, BX, BY, BZ, CA, CB, CC |
| **AA** | <1:3 |  | 81.81 | A, B, C, D, E, F, G, H, I, J, K, L, M, O, P, Q, R, S, U, V, W, X, Y, Z, AC, AE, AF, AG, AJ, AM, AN, AO, AQ, AR, AT, AU, AV, AW, AX, AY, AZ, BA, BB, BC, BD, BE, BF, BG, BH, BI, BJ, BK, BL, BM, BP, BQ, BR, BS, BT, BU, BV, BW, BX, BY, BZ, CA, CB, CC |  | 77.23 | A, B, C, D, E, F, G, H, I, J, K, L, M, N, O, P, Q, R, S, U, V, X, Y, Z, AB, AC, AE, AF, AG, AJ ,AK, AL, AM, AN, AO, AP, AQ, AR, AT, AU, AV, AW, AX, AY, AZ, BA, BB, BC, BD, BE, BF, BG, BH, BI, BJ, BL, BM, BN, BO, BP, BQ, BR, BS, BT, BU, BV, BX, BY, BZ, CA, CB, CC |
| **AB** | <1:3 |  | 84.96 | A, B, C, D, E, F, G, H, I, J, K, L, M, O, P, Q, R, S, U, V, W, X, Y, Z, AC, AD, AE, AF, AG, AH, AJ, AL, AM, AN, AO, AQ, AR, AS, AT, AU, AV, AW, AX, AY, AZ, BA, BB, BC, BD, BE, BF, BG, BH, BI, BJ, BK, BL, BM, BP, BQ, BR, BS, BT, BU, BV, BW, BX, BY, BZ, CA, CB, CC |  | 97.86 | B, D, E, F, G, J, L, M, N, Q, R, S, U, V, W, Z, AA, AD, AE, AF, AG, AH, AI, AK, AM, AN, AP, AQ, AS, AT, AU, AV, AW, AX, AY, AZ, BA, BB, BC, BD, BE, BG, BH, BJ, BK, BL, BM, BQ, BR, BS, BV, BW, BX, BY, BZ, CA, CB, CC |
| **AC** | >1:12 |  | 95.59 | A, B, C, E, F, G, H, I, J, K, L, N, P, Q, R, S, T, V, W, Y, Z, AA, AB, AD, AE, AF, AG, AH, AI, AK, AL, AM, AN, AO, AP, AS, AT, AV, AX, AY, BA, BB, BD, BE, BF, BH, BI, BJ, BL, BN, BP, BQ, BR, BS, BU, BW, BX, BY, BZ, CA, CB, CC |  | 98.02 | B, D, E, F, G, J, L, M, Q, R, S, U, V, W, Z, AA, AB, AD, AE, AF, AG, AH, AI ,AK, AM, AN, AP, AQ, AS, AT, AU, AV, AW, AX, AY, BA, BB, BC, BD, BE, BG, BH, BJ, BK, BL, BM, BQ, BR, BS, BV, BW, BX, BY, BZ, CA, CB, CC |
| **AD** | <1:3 |  | 71.36 | B, C, D, E, F, G, H, K, R, S, U, V, X, Y, AB, AC, AE, AF, AH, AJ, AR, AS, AT, AU, AV, AW, AX, AY, AZ, BA, BB, BC, BD, BE, BF, BH, BJ, BK, BL, BM, BR, BS, BT, BU, BW, BX, BY, BZ, CA, CB, CC |  | 64.68 | A, C, D, E, F, G, H, I, J, K, M, N, O, P, Q, R, S, U, V, X, Y, Z, AB, AC, AE, AG, AJ, AL, AM, AN, AO, AP, AQ, AR, AU, AW, AY, AZ, BC, BD, BE, BF, BG, BH, BI, BJ, BL, BM, BN, BO, BP, BQ, BR, BS, BT, BU, BV, BZ, CA, CB, CC |
| **AE** | <1:3 |  | 31.94 | A, C, D, E, F, G, H, I, J, K, L, M, N, O, P, Q, R, S, T, U, V, W, X, Y, Z, AA, AB, AC, AD, AG, AH, AI, AJ, AK, AL, AM, AN, AO, AP, AQ, AR, AS, AT, AU, AV, AW, AX, AY, AZ, BA, BB, BC, BD, BE, BF, BG, BH, BI, BJ, BK, BL, BM, BN, BO, BP, BQ, BR, BS, BT, BU, BV, BW, BX, BY, BZ, CA, CB, CC |  | 13.12 | A, B, C, D, E, F, G, H, I, J, K, L, M, N, O, P, Q, R, S, T, U, V, W, X, Y, Z, AA, AB, AC, AD, AF, AG, AH, AI, AJ ,AK, AL, AM, AN, AO, AP, AQ, AR, AS, AT, AU, AV, AW, AX, AY, AZ, BA, BB, BC, BD, BE, BF, BG, BH, BI, BJ, BK, BL, BM, BN, BO, BP, BQ, BR, BS, BT, BU, BV, BW, BX, BY, BZ, CA, CB, CC |
| **AF** | <1:3 |  | 40.56 | A, C, D, E, F, G, H, I, J, K, L, M, N, O, P, Q, R, T, U, V, W, X, Y, Z, AA, AB, AC, AD, AG, AH, AI, AJ, AK, AL, AM, AN, AO, AP, AQ, AR, AS, AT, AU, AV, AW, AX, AY, AZ, BA, BC, BD, BE, BF, BG, BH, BI, BJ, BK, BL, BM, BN, BO, BP, BQ, BR, BS, BT, BU, BV, BZ, CA, CB, CC |  | 49.51 | A, C, D, E, F, G, H, I, J, K, M, N, O, P, Q, R, T, U, V, X, Y, Z, AA, AB, AC, AE, AG, AH, AI, AJ, AL, AM, AN, AO, AP, AQ, AR, AS, AU, AW, AY, AZ, BC, BD, BE, BF, BG, BH, BI, BJ, BK, BL, BM, BN, BO, BP, BQ, BR, BS, BT, BU, BV, BZ, CA, CB, CC |
| **AG** | >1:12 |  | 87.42 | B, C, D, E, F, G, H, I, J, K, L, M, O, Q, R, S, U, V, W, X, Y, Z, AA, AB, AC, AE, AF, AH, AJ, AK, AL, AN, AQ, AR, AS, AT, AU, AV, AW, AX, AY, AZ, BA, BB, BC, BD, BE, BF, BG, BH, BI, BJ, BK, BL, BM, BN, BP, BR, BS, BT, BU, BV, BW, BX, BY, BZ, CA, CB, CC |  | 99.95 | A, B, C, H, I,K, L, M, N, P, R, S, T, U, W, X, Y, Z, AA, AB, AC, AD, AE, AF, AH, AI, AJ ,AK, AL, AM, AN, AO, AP, AR, AS, AT, AU, AV, AW, AX, AY, AZ, BA, BB, BF, BI, BJ, BK, BL, BP, BT, BU, BV, BW, BX, BY |
| **AH** | >1:12 |  | 71.44 | A, B, C, D, E, F, G, H, I, J, K, L, M, N, O, P, Q, R, S, T, U, V, W, X, Y, Z, AB, AC, AD, AE, AF, AG, AI, AJ, AK, AL, AM, AN, AO, AP, AQ, AR, AT, AU, AV, AW, AY, AZ, BB, BC, BD, BE, BF, BG, BH, BI, BJ, BK, BL, BM, BP, BQ, BR, BS, BT, BU, BV, BW, BX, BY, BZ, CA, CB, CC |  | 80.22 | A, B, C, D, E, F, G, H, I, J, K, L, M, N, O, P, Q, R, S,U, V, W, X, Y, Z, AB, AC, AE, AF, AG, AJ, AL, AM, AN, AO, AP, AQ, AR, AT, AU, AV, AW, AX, AY, AZ, BA, BB, BC, BD, BE, BF, BG, BH, BI, BJ,BL, BM, BN, BO, BP, BQ, BR, BS, BT, BU, BV, BW, BX, BY, BZ, CA, CB, CC |
| **AI** | <1:3 |  | 83.50 | A, B, C, D, E, F, G, H, I, J, K, L, M, O, P, Q, R, S, U, V, W, X, Y, Z, AC, AE, AF, AH, AJ, AN, AQ, AR, AS, AT, AU, AV, AW, AX, AY, AZ, BA, BB, BC, BD, BE, BF, BG, BH, BI, BJ, BK, BL, BM, BP, BR, BS, BT, BU, BV, BW, BX, BY, BZ, CA, CB, CC |  | 86.96 | A, B, C, D, E, F, G, H, I, J, K, L, M, N, O, P, Q, S, U, V, W, X, Y, Z, AB, AC, AE, AF, AG, AJ ,AK, AL, AN, AO, AQ, AR, AS, AT, AV, AX, AZ, BA, BB, BC, BD, BE, BF, BG, BH, BI, BJ, BM, BN, BO, BP, BQ, BR, BS, BT, BU, BV, BW, BX, BY, BZ, CA, CB, CC |
| **AJ** | >1:12 |  | 95.07 | A, B, C, E, F, G, H, I, J, L, N, P, Q, R, S, T, V, W, Y, Z, AA, AB, AD, AE, AF, AG, AH, AI, AK, AL, AM, AN, AO, AP, AS, AT, AV, AX, AY, BA, BB, BD, BE, BF, BG, BH, BI, BN, BP, BQ, BS, BU, BV, BW, BX, BY, BZ, CA, CB |  | 98.91 | B, D, E, F, G, L, M, N, P, Q, T, V, W, Z, AA, AD, AE, AF, AG, AH, AI, AK, AM, AN, AO, AP, AS, AT, AU, AV, AW, AX, AY, AZ, BA, BB, BC, BD, BF, BG, BJ, BK, BL, BQ, BS, BT, BU, BV, BW, BX, BY, BZ, CA, CB, CC |
| **AK** | <1:3 |  | 82.65 | A, B, C, D, E, F, G, H, I, J, K, L, M, O, P, Q, R, S, U, V, W, X, Y, Z, AC, AE, AF, AG, AH, AJ, AM, AN, AO, AQ, AR, AS, AT, AU, AV, AW, AX, AY, AZ, BA, BB, BC, BD, BE, BF, BG, BH, BI, BJ, BK, BL, BM, BP, BQ, BR, BS, BT, BU, BV, BW, BX, BY, BZ, CA, CB, CC |  | 58.02 | A, C, D, E, F, G, H, I, J, K, M, N, O, P, Q, R, S, T, U, V, X, Y, Z, AA, AB, AC, AE, AG, AH, AI, AJ, AL, AM, AN, AO, AP, AQ, AR, AU, AW, AY, AZ, BB, BC, BD, BE, BF, BG, BH, BI, BJ, BK, BL, BM, BN, BO, BP, BQ, BR, BS, BT, BU, BV, BZ, CA, CB, CC |
| **AL** | <1:3 |  | 82.45 | A, B, C, D, E, F, G, H, I, J, K, L, M, N, O, P, Q, R, S, T, U, V, W, X, Y, Z, AB, AC, AE, AF, AG, AH, AJ, AM, AN, AO, AQ, AR, AS, AT, AU, AV, AW, AX, AY, AZ, BA, BB, BC, BD, BE, BF, BG, BH, BI, BJ, BK, BL, BM, BP, BQ, BR, BS, BT, BU, BV, BW, BX, BY, BZ, CA, CB, CC |  | 98.67 | B, D, E, F, G, J, L, M, N, Q, R, S, T, V, W, Z, AA, AD, AE, AF, AG, AH, AI, AK, AM, AN, AP, AS, AT, AU, AV, AW, AX, AY, AZ, BA, BB, BC, BD, BF, BG, BH, BJ, BK, BL, BM, BQ, BR, BS, BT, BV, BW, BX, BY, BZ, CA, CB, CC |
| **AM** | <1:3 |  | 88.54 | B, C, D, E, F, G, H, I, J, K, L, M, O, R, S, U, V, W, X, Y, Z, AA, AB, AC, AE, AF, AH, AJ, AK, AL, AQ, AR, AS, AT, AU, AV, AW, AX, AY, AZ, BA, BB, BC, BD, BE, BF, BG, BH, BI, BJ, BK, BL, BM, BN, BP, BR, BS, BT, BU, BV, BW, BX, BY, BZ, CA, CB, CC |  | 92.26 | A, B, C, D, E, F, G, H, I, J, K, L, N, O, P, Q, S, U, V, W, Y, AA, AB, AC, AD, AE, AF, AG, AH, AJ, AK, AL, AN, AO, AQ, AR, AS, AT, AV, AX, BA, BB, BC, BD, BE, BF, BG, BH, BI, BJ, BK, BM, BN, BO, BP, BQ, BR, BS, BT, BU, BW, BX, BY, BZ, CA, CB, CC |
| **AN** | >1:12 |  | 89.90 | B, C, D, E, F, G, H, I, K, L, M, R, S, T, U, V, W, X, Y, AA, AB, AC, AE, AF, AG, AH, AI, AJ, AK, AL, AP, AQ, AR, AS, AT, AU, AV, AW, AX, AY, AZ, BA, BB, BC, BD, BE, BF, BG, BH, BJ, BK, BL, BM, BN, BP, BR, BS, BT, BU, BV, BW, BX, BY, BZ, CA, CB, CC |  | 96.47 | A, B, D, E, F, G, H, I, J, K, L, O, Q, S, T, V, W, AA, AB, AC, AD, AE, AF, AG, AH, AI, AJ, AK, AL, AM, AO, AP, AQ, AR, AS, AT, AV, AW, AX, BA, BB, BC, BD, BE, BG, BH, BI, BK, BL, BM, BN, BQ, BR, BS, BT, BU, BV, BW, BX, BY, BZ, CA, CB, CC |
| **AO** | >1:12 |  | 89.80 | B, C, D, E, F, G, H, K, L, M, O, R, S, U, V, W, X, Y, AA, AB, AC, AE, AF, AH, AJ, AK, AL, AP, AQ, AR, AS, AT, AU, AV, AW, AX, AY, AZ, BA, BB, BC, BD, BE, BF, BG, BH, BI, BJ, BK, BL, BM, BN, BR, BS, BT, BU, BW, BX, BY, BZ, CA, CB, CC |  | 98.06 | B, D, E, F, G, J, K, L, M, N, Q, R, S, T, V, W, Z, AA, AD, AE, AF, AG, AH, AI, AJ, AK, AM, AN, AP, AQ, AS, AT, AU, AV, AW, AX, AY, BA, BB, BC, BD, BG, BH, BI, BJ, BK, BL, BM, BQ, BR, BS, BT, BV, BW, BX, BY, BZ, CA, CB, CC |
| **AP** | >1:12 |  | 84.47 | A, B, C, D, E, F, G, H, I, J, K, L, M, O, P, Q, R, S, U, V, W, X, Y, Z, AC, AE, AF, AH, AJ, AN, AO, AP, AQ, AR, AS, AT, AU, AV, AW, AX, AY, AZ, BA, BB, BC, BD, BE, BF, BG, BH, BI, BJ, BK, BL, BM, BP, BR, BS, BT, BU, BV, BW, BX, BY, BZ, CA, CB, CC |  | 94.54 | A, B, C, D, E, F, G, H, I, J, K, L, N, O, Q, S, U, V, W, X, Y, AA, AB, AC, AD, AE, AF, AG, AH, AJ, AK, AL, AN, AO, AQ, AR, AS, AT, AV, AX, AZ, BA, BB, BC, BD, BE, BF, BG, BH, BI, BJ, BK, BM, BN, BQ, BR, BS, BT, BU, BW, BX, BY, BZ, CA, CB, CC |
| **AQ** | >1:12 |  | 94.97 | A, B, C, E, F, G, H, I, J, K, L, N, P, R, S, T, V, W, Y, Z, AA, AB, AE, AF, AG, AH, AI, AK, AL, AM, AN, AO, AP, AS, AT, AV, AX, AY, BA, BB, BD, BE, BF, BH, BI, BJ, BL, BM, BN, BP, BQ, BR, BS, BU, BW, BX, BY, BZ, CA, CB, CC |  | 91.13 | B, E, F, G, H, I, J, K, L, M, N, P, R, S, T, V, W, X, Z, AA, AB, AC, AD, AE, AF, AH, AI, AK, AM, AN, AO, AP, AR, AS, AT, AU, AV, AW, AX, AY, AZ, BA, BB, BF, BG, BJ, BK, BL, BS, BT, BU, BV, BW, BX, BY, BZ, CB, CC |
| **AR** | >1:12 |  | 95.99 | A, B, C, E, F, G, H, I, J, L, N, P, Q, R, S, T, V, W, Y, Z, AA, AC, AD, AE, AF, AG, AH, AI, AK, AL, AM, AN, AO, AP, AS, AT, AV, AX, AY, BA, BB, BD, BE, BF, BG, BH, BI, BJ, BL, BN, BP, BQ, BR, BS, BU, BV, BW, BX, BY, BZ, CA, CB |  | 96.53 | B, D, E, F, G, J, L, M, N, Q, R, S, T, V, W, Z, AA, AD, AE, AF, AG, AH, AI, AK, AM, AN, AP, AQ, AS, AT, AU, AV, AW, AX, AY, AZ, BA, BB, BC, BD, BE, BG, BH, BJ, BK, BL, BM, BQ, BR, BS, BV, BW, BX, BY, BZ, CA, CB, CC |
| **AS** | >1:12 |  | 77.15 | A, B, C, D, E, F, G, H, I, J, K, L, M, N, O, P, Q, R, S, T, U, V, W, X, Y, Z, AB, AC, AD, AE, AF, AG, AI, AJ, AK, AL, AM, AN, AO, AP, AQ, AR, AT, AU, AV, AW, AX, AY, AZ, BA, BB, BC, BD, BE, BF, BG, BH, BI, BJ, BK, BL, BM, BP, BQ, BR, BS, BT, BU, BV, BW, BX, BY, BZ, CA, CB, CC |  | 77.76 | A, B, C, D, E, F, G, H, I, J, K, L, M, N, O, P, Q, R, S, T, U, V, W, X, Y, Z, AB, AC, AE, AF, AG, AI, AJ, AL, AM, AN, AO, AP, AQ, AR, AT, AU, AV, AW, AX, AY, AZ, BA, BB, BC, BD, BE, BF, BG, BH, BI, BJ, BL, BM, BN, BO, BP, BQ, BR, BS, BT, BU, BV, BW, BX, BY, BZ, CA, CB, CC |
| **AT** | <1:3 |  | 51.70 | A, B, C, D, E, F, G, H, I, J, K, L, M, N, O, P, Q, R, T, U, V, X, Y, Z, AA, AB, AC, AD, AE, AF, AG, AH, AI, AJ, AK, AL, AM, AN, AO, AP, AQ, AR, AS, AU, AW, AX, AY, AZ, BA, BC, BD, BE, BF, BG, BH, BI, BJ, BK, BL, BM, BN, BO, BP, BQ, BR, BS, BT, BU, BV, BW, BX, BZ, CA, CB, CC |  | 42.47 | A, C, D, E, F, G, H, I, J, K, M, N, O, P, Q, R, S, T, U, V, W, X, Y, Z, AA, AB, AC, AE, AG, AH, AI, AJ, AL, AM, AN, AO, AP, AQ, AR, AS, AU, AW, AY, AZ, BA, BC, BD, BE, BF, BG, BH, BI, BJ, BK, BL, BM, BN, BO, BP, BQ, BR, BS, BT, BU, BV, BW, BX, BY, BZ, CA, CB, CC |
| **AU** | <1:6 |  | 95.19 | A, B, C, E, F, G, H, J, K, L, N, P, R, S, T, V, W, Y, Z, AA, AB, AD, AE, AF, AG, AH, AI, AK, AL, AM, AN, AO, AP, AS, AT, AV, AX, AY, BA, BB, BD, BE, BF, BH, BJ, BL, BN, BQ, BR, BS, BU, BW, BX, BY, BZ, CA, CB |  | 92.99 | A, B, C, D, E, F, G, H, I, J, K, L, N, O, Q, S, U, V, W, X, Y, AA, AB, AC, AD, AE, AF, AG, AH, AJ, AK, AL, AO, AQ, AR, AS, AT, AV, AX, BA, BB, BC, BD, BE, BF, BG, BH, BI, BK, BM, BN, BO, BQ, BR, BS, BU, BW, BX, BY, BZ, CA, CB, CC |
| **AV** | <1:3 |  | 51.57 | A, B, C, D, E, F, G, H, I, J, K, L, M, N, O, P, Q, R, T, U, V, X, Y, Z, AA, AB, AC, AD, AE, AF, AG, AH, AI, AJ, AK, AL, AM, AN, AO, AP, AQ, AR, AS, AU, AW, AX, AY, AZ, BA, BC, BD, BE, BF, BG, BH, BI, BJ, BK, BL, BM, BN, BO, BP, BQ, BR, BS, BT, BU, BV, BW, BZ, CA, CB, CC |  | 39.03 | A, C, D, E, F, G, H, I, J, K, M, N, O, P, Q, R, T, U, V, W, X, Y, Z, AA, AB, AC, AE, AG, AH, AI, AJ, AL, AM, AN, AO, AP, AQ, AR, AS, AU, AW, AY, AZ, BA, BC, BD, BE, BF, BG, BH, BI, BJ, BK, BL, BM, BN, BO, BP, BQ, BR, BS, BT, BU, BV, BW, BX, BY, BZ, CA, CB, CC |
| **AW** | <1:3 |  | 98.07 | A, B, E, G, I, J, L, N, P, Q, R, S, T, V, W, Y, Z, AA, AB, AD, AE, AF, AG, AH, AI, AK, AL, AM, AN, AO, AP, AS, AT, AV, AX, AY, BA, BB, BD, BE, BG, BH, BI, BJ, BN, BO, BP, BQ, BV, BW, BX, BY, CA |  | 91.73 | A, B, C, D, E, F, G, H, I, J, K, L, M, N, O, P, Q, S, U, V, W, X, Y, Z, AA, AB, AC, AD, AE, AF, AG, AH, AJ, AK, AL, AN, AO, AQ, AR, AS, AT, AV, AX, AY, AZ, BA, BB, BC, BD, BE, BF, BG, BH, BI, BJ, BK, BM, BN, BO, BP, BQ, BR, BS, BT, BU, BV, BW, BX, BY, BZ, CA, CB, CC |
| **AX** | <1:3 |  | 65.87 | A, B, C, D, E, F, G, H, I, J, K, M, N, O, P, Q, R, S, T, U, V, W, X, Y, Z, AA, AB, AC, AD, AE, AF, AG, AI, AJ, AK, AL, AM, AN, AO, AP, AQ, AR, AS, AT, AU, AV, AW, AY, AZ, BB, BC, BD, BE, BF, BG, BH, BI, BJ, BK, BL, BM, BN, BP, BQ, BR, BS, BT, BU, BV, BW, BX, BY, BZ, CA, CB, CC |  | 47.73 | A, C, D, E, F, G, H, I, J, K, M, N, O, P, Q, R, S, T, U, V, W, X, Y, Z, AA, AB, AC, AE, AG, AH, AI, AJ, AL, AM, AN, AO, AP, AQ, AR, AS, AU, AW, AY, AZ, BB, BC, BD, BE, BF, BG, BH, BI, BJ, BK, BL, BM, BN, BO, BP, BQ, BR, BS, BT, BU, BV, BW, BZ, CA, CB, CC |
| **AY** | >1:12 |  | 99.77 | A, B, D, H, I, J, K, L, M, N, O, P, Q, S, T, U, V, W, X, Y, Z, AA, AB, AC, AD, AE, AG, AH, AJ, AK, AL, AM, AN, AO, AP, AQ, AR, AS, AT, AU, AV, AW, AX, AZ, BA, BB, BC, BG, BI, BK, BL, BM, BN, BO, BP, BQ, BR, BT, BV, BW, BX, BY, BZ, CB |  | 94.72 | A, B, C, D, E, F, G, H, I, J, K, L, N, O, Q, S, U, V, W, Y, AA, AB, AC, AD, AE, AF, AG, AH, AJ, AK, AL, AO, AQ, AR, AS, AT, AV, AW, AX, BA, BB, BC, BD, BE, BG, BH, BI, BK, BL, BM, BN, BO, BQ, BR, BS, BU, BW, BX, BY, BZ, CA, CB, CC |
| **AZ** | <1:3 |  | 96.33 | A, B, C, E, F, G, H, I, J, L, N, P, Q, R, S, T, V, W, Y, Z, AA, AB, AD, AE, AF, AG, AH, AI, AK, AL, AM, AN, AO, AP, AS, AT, AV, AX, AY, BA, BB, BD, BE, BF, BH, BI, BJ, BN, BP, BQ, BS, BU, BV, BW, BX, BY, BZ, CA, CB |  | 95.91 | A, B, D, E, F, G, J, K, L, O, Q, S, T, V, W, AA, AB, AD, AE, AF, AG, AH, AI, AJ, AK, AL, AP, AQ, AR, AS, AT, AV, AW, AX, BA, BB, BC, BD, BE, BG, BH, BI, BK, BL, BM, BN, BQ, BR, BS, BW, BX, BY, BZ, CA, CB, CC |
| **BA** | <1:3 |  | 64.68 | A, B, C, D, E, F, G, H, I, J, K, M, N, O, P, Q, R, S, T, U, V, W, X, Y, Z, AA, AB, AC, AD, AE, AF, AG, AI, AJ, AK, AL, AM, AN, AO, AP, AQ, AR, AS, AT, AU, AV, AW, AY, AZ, BB, BC, BD, BE, BF, BG, BH, BI, BJ, BK, BL, BM, BN, BP, BQ, BR, BS, BT, BU, BV, BW, BX, BY, BZ, CA, CB, CC |  | 61.16 | A, B, C, D, E, F, G, H, I, J, K, M, N, O, P, Q, R, S, T, U, V, X, Y, Z, AA, AB, AC, AE, AG, AH, AI, AJ, AL, AM, AN, AO, AP, AQ, AR, AS, AT, AU, AV, AW, AY, AZ, BB, BC, BD, BE, BF, BG, BH, BI, BJ, BK, BL, BM, BN, BO, BP, BQ, BR, BS, BT, BU, BV, BZ, CA, CB, CC |
| **BB** | <1:3 |  | 46.22 | A, B, C, D, E, F, G, H, I, J, K, L, M, N, O, P, Q, R, T, U, V, X, Y, Z, AA, AB, AC, AD, AE, AG, AH, AI, AJ, AK, AL, AM, AN, AO, AP, AQ, AR, AS, AU, AW, AX, AY, AZ, BA, BC, BD, BE, BF, BG, BH, BI, BJ, BK, BL, BM, BN, BO, BP, BQ, BR, BS, BT, BU, BV, BZ, CA, CB, CC |  | 34.84 | A, C, D, E, F, G, H, I, J, K, M, N, O, P, Q, R, T, U, V, X, Y, Z, AA, AB, AC, AE, AG, AH, AI, AJ, AL, AM, AN, AO, AP, AQ, AR, AS, AU, AW, AY, AZ, BC, BD, BE, BF, BG, BH, BI, BJ, BK, BL, BM, BN, BO, BP, BQ, BR, BS, BT, BU, BV, BW, BX, BY, BZ, CA, CB, CC |
| **BC** | >1:12 |  | 96.42 | A, B, C, E, F, G, H, I, J, L, N, P, Q, R, S, T, V, W, Y, Z, AA, AB, AD, AE, AF, AG, AH, AI, AK, AL, AM, AN, AO, AP, AS, AT, AV, AX, AY, BA, BB, BD, BE, BF, BG, BH, BI, BJ, BN, BP, BQ, BS, BU, BV, BW, BX, BY, BZ, CA, CB |  | 99.96 | A, B, C, H, I, K, L, M, N, P, R, S, T, U, W, X, Y, Z, AA, AB, AC, AD, AE, AF, AH, AI, AJ, AK, AL, AM, AN, AO, AP, AR, AS, AT, AU, AV, AW, AX, AY, AZ, BA, BB, BF, BI, BJ, BK, BL, BO, BP, BR, BT, BU, BV, BW, BX, BY |
| **BD** | >1:12 |  | 100.00 | A, B, D, H, I, J, K, L, M, N, O, P, Q, S, T, U, W, X, Z, AA, AB, AC, AD, AE, AF, AG, AH, AI, AJ, AK, AL, AM, AN, AO, AP, AQ, AR, AS, AT, AU, AV, AW, AX, AZ, BA, BB, BC, BF, BG, BI, BK, BL, BM, BN, BO, BP, BQ, BR, BT, BU, BV, BW, BX, BY, BZ, CB, CC |  | 99.91 | A, B, C, H, I, K, L, M, N, P, R, S, T, U, W, X, Y, Z, AA, AB, AC, AD, AE, AF, AH, AI, AJ, AK, AL, AM, AN, AO, AP, AR, AS, AT, AU, AV, AW, AX, AY, AZ, BA, BB, BF, BI, BJ, BK, BL, BP, BT, BU, BV, BW, BX, BY |
| **BE** | >1:12 |  | 99.98 | A, B, D, H, I, J, K, L, M, N, O, P, Q, S, T, U, W, X, Z, AA, AB, AC, AD, AE, AF, AG, AH, AI, AJ, AK, AL, AM, AN, AO, AP, AQ, AR, AS, AT, AU, AV, AW, AX, AZ, BA, BB, BC, BF, BG, BI, BK, BL, BM, BN, BO, BP, BQ, BR, BT, BU, BV, BW, BX, BY, BZ, CB, CC |  | 96.82 | B, E, F, G, L, M, N, P, R, S, T, V, W, X, Z, AA, AB, AC, AD, AE, AF, AH, AI, AK, AM, AN, AP, AR, AS, AT, AU, AV, AW, AX, AY, AZ, BA, BB, BF, BG, BJ, BK, BL, BS, BT, BU, BV, BW, BX, BY, BZ, CB, CC |
| **BF** | >1:12 |  | 98.99 | A, B, D, E, G, I, J, L, M, N, O, P, Q, R, S, T, V, W, X, Z, AA, AB, AC, AD, AE, AF, AG, AH, AI, AJ, AK, AL, AM, AN, AO, AP, AQ, AR, AS, AT, AU, AV, AX, AZ, BA, BB, BC, BD, BE, BG, BH, BI, BJ, BK, BM, BN, BO, BP, BQ, BV, BW, BX, BY |  | 96.70 | B, D, E, F, G, J, K, L,Q, S, T, V, W, AA, AD, AE, AF, AG, AH, AI, AJ, AK, AL, AM, AP, AQ, AS, AT, AU, AV, AW, AX, BA, BB, BC, BD, BE, BG, BH, BI, BK, BL, BM, BN, BQ, BR, BS, BW, BX, BY, BZ, CA, CB, CC |
| **BG** | >1:12 |  | 94.63 | A, B, C, E, F, G, H, I, J, K, L, N, P, R, S, T, V, W, Y, Z, AA, AB, AE, AF, AG, AH, AI, AJ, AK, AL, AM, AN, AO, AP, AR, AS, AT, AV, AW, AX, AY, BA, BB, BC, BD, BE, BF, BH, BI, BJ, BL, BM, BN, BQ, BR, BS, BU, BW, BX, BY, BZ, CA, CB, CC |  | 100.00 | A, B, C, H, I, K, L, M, N, P, R, S, T, U, W, X, Y, Z, AA, AB, AC, AD, AE, AF, AH, AI, AJ, AK, AL, AM, AN, AO, AP, AQ, AR, AS, AT, AU, AV, AW, AX, AY, AZ, BA, BB, BE, BF, BI, BJ, BK, BL, BM, BO, BP, BR, BT, BU, BV, BW, BX, BY |
| **BH** | >1:12 |  | 99.95 | A, B, D, H, I, J, K, L, M, N, O, P, Q, S, T, U, W, X, Z, AA, AB, AC, AD, AE, AF, AG, AH, AI, AJ, AK, AL, AM, AN, AO, AP, AQ, AR, AS, AT, AU, AV, AW, AX, AZ, BA, BB, BC, BF, BG, BI, BK, BL, BM, BN, BO, BP, BQ, BR, BT, BU, BV, BW, BX, BY, BZ, CB, CC |  | 99.66 | A, B, C, I, K, L, M, N, P, R, S, T, U, W, X, Y, AA, AB, AC, AD, AE, AF, AH, AI, AK, AL, AM, AN, AO, AP, AR, AS, AT, AU, AV, AW, AX, AY, AZ, BA, BB, BF, BJ, BK, BL, BP, BT, BU, BV, BW, BX, BY |
| **BI** | >1:12 |  | 92.99 | A, B, C, D, E, F, G, H, J, K, L, N, R, S, T, U, V, W, X, Y, Z, AA, AB, AC, AE, AF, AG, AH, AI, AJ, AK, AL, AM, AP, AQ, AR, AS, AT, AV, AW, AX, AY, AZ, BA, BB, BC, BD, BE, BF, BG, BH, BJ, BK, BL, BM, BN, BQ, BR, BT, BU, BW, BX, BY, BZ, CA, CB, CC |  | 98.22 | B, D, E, F, G, L, M, N, Q, R, S, T, V, Y, Z, AA, AD, AE, AF, AG, AH, AI, AK, AM, AN, AO, AP, AS, AT, AU, AV, AW, AX, AY, AZ, BA, BB, BC, BD, BJ, BK, BL, BM, BQ, BS, BT, BV, BW, BX, BY, BZ, CA, CB, CC |
| **BJ** | >1:12 |  | 100.00 | A, B, D, H, I, J, K, L, M, N, O, P, Q, S, T, U, W, X, Z, AA, AB, AC, AD, AE, AF, AG, AH, AI, AJ, AK, AL, AM, AN, AO, AP, AQ, AR, AS, AT, AU, AV, AW, AX, AZ, BA, BB, BC, BF, BG, BI, BJ, BK, BL, BM, BN, BO, BP, BQ, BR, BT, BU, BV, BW, BX, BY, BZ, CB, CC |  | 96.31 | A, B, D, E, F, G, H, J, K, L, O, Q, S, T, V, W, AA, AB, AC, AD, AE, AF, AG, AH, AI, AJ, AK, AL, AM, AO, AP, AQ, AR, AS, AT, AV, AW, AX, BA, BB, BC, BD, BE, BG, BH, BI, BK, BL, BM, BN, BQ, BR, BS, BW, BX, BY, BZ, CA, CB, CC |
| **BK** | <1:3 |  | 95.39 | A, B, C, E, F, G, H, I, J, L, N, P, Q, R, S, T, V, W, Y, Z, AA, AB, AD, AE, AF, AG, AH, AJ, AK, AL, AM, AN, AO, AP, AS, AT, AV, AX, AY, BA, BB, BD, BE, BF, BH, BI, BJ, BL, BN, BQ, BR, BS, BU, BW, BX, BY, BZ, CA, CB |  | 83.67 | A, B, C, D, E, F, G, H, I, J, K, L, M, N, O, P, Q, R, S, U, V, W, X, Y, Z, AB, AC, AE, AF, AG, AJ, AK, AL, AM, AN, AO, AP, AQ, AR, AT, AU, AV, AW, AX, AY, AZ, BA, BB, BC, BD, BE, BF, BG, BH, BI, BJ,BL, BM, BN, BO, BP, BQ, BR, BS, BT, BU, BV, BW, BX, BY, BZ, CA, CB, CC |
| **BL** | >1:12 |  | 98.01 | A, B, D, E, G, I, J, L, M, N, O, P, Q, R, S, T, V, W, X, Y, Z, AA, AB, AC, AD, AE, AF, AG, AH, AI, AK, AL, AM, AN, AO, AP, AQ, AR, AS, AT, AU, AV, AX, AY, BA, BB, BD, BE, BG, BH, BI, BJ, BK, BM, BN, BO, BP, BQ, BV, BW, BX, BY |  | 92.99 | A, B, C, D, E, F, G, H, I, J, K, L, M, N, O, P, Q, R, S, U, V, W, X, Y, Z, AA, AB, AC, AD, AE, AF, AG, AH, AJ, AK, AL AN, AO, AQ, AR, AS, AT, AV, AX, AY, AZ, BA, BB, BC, BD, BE, BF, BG, BH, BI, BJ, BK, BM, BN, BP, BQ, BR, BS, BT, BU, BV, BW, BX, BY, BZ, CA, CB, CC |
| **BM** | <1:3 |  | 97.02 | A, B, C, E, F, G, I, J, L, N, P, Q, R, S, T, V, W, Y, Z, AA, AB, AD, AE, AF, AG, AH, AI, AK, AL, AM, AN, AO, AP, AQ, AS, AT, AV, AX, AY, BA, BB, BD, BE, BF, BG, BH, BI, BJ, BN, BP, BQ, BS, BV, BW, BX, BY, BZ, CA |  | 99.77 | A, B, C, E, F, I, K, L, M, N, P, R, S, T, V, W, X, Z, AA, AB, AC, AD, AE, AF, AH, AI, AK, AL, AM, AN, AO, AP, AR, AS, AT, AU, AV, AW, AX, AY, AZ, BA, BB, BF, BG, BI, BJ, BK, BL, BP, BS, BT, BU, BV, BW, BX, BY, BZ, CB, CC |
| **BN** | >1:12 |  | 78.52 | A, B, C, D, E, F, G, H, I, J, K, L, M, N, O, P, Q, R, S, U, V, W, X, Y, Z, AC, AE, AF, AG, AJ, AM, AN, AO, AQ, AR, AT, AU, AV, AW, AX, AY, AZ, BA, BB, BC, BD, BE, BF, BG, BH, BI, BJ, BK, BL, BM, BP, BQ, BR, BS, BT, BU, BV, BW, BX, BY, BZ, CA, CB, CC |  | 98.19 | B, L, M, N, P, R, S, T, W, X, Z, AA, AD, AE, AF, AH, AI, AK, AM, AN, AP, AS, AT, AU, AV, AW, AX, AY, AZ, BA, BB, BF, BJ, BK, BL, BT, BV, BW, BX, BY |
| **BO** | <1:3 |  | 73.34 | B, C, E, F, G, H, K, R, S, V, Y, AE, AF, AT, AV, AW, AY, BB, BD, BE, BF, BH, BJ, BL, BR, BS, BT, BU, BW, BX, BY, BZ, CA, CB, CC |  | 92.01 | B, E, F, G, L, M, Q, S, T, V, W, AA, AD, AE, AF, AH, AI, AK, AM,AS, AT, AU, AV, AW, AX, AY, BA, BB, BC, BG, BK, BS, BW, BX, BY, BZ, CA, CB, CC |
| **BP** | <1:3 |  | 93.60 | A, B, C, D, E, F, G, H, J, K, L, N, Q, R, S, T, U, V, W, X, Y, Z, AA, AB, AC, AE, AF, AG, AH, AI, AJ, AK, AL, AM, AN, AP, AQ, AR, AS, AT, AV, AW, AX, AY, AZ, BA, BB, BC, BD, BE, BF, BH, BJ, BL, BM, BN, BQ, BR, BS, BT, BU, BW, BX, BY, BZ, CA, CB, CC |  | 95.63 | B, D, E, F, G, J, L, Q, S, T, V, W, AA, AD, AE, AF, AG, AH, AI, AK, AM, AS, AT, AV, AW, AX, BA, BB, BC, BD, BG, BH, BK, BL, BM, BQ, BR, BS, BW, BX, BY, BZ, CA, CB, CC |
| **BQ** | <1:6 |  | 88.49 | B, C, D, E, F, G, H, I, K, L, M, O, R, S, U, V, W, X, Y, AA, AB, AC, AE, AF, AH, AJ, AK, AL, AQ, AR, AS, AT, AU, AV, AW, AX, AY, AZ, BA, BB, BC, BD, BE, BF, BG, BH, BI, BJ, BK, BL, BM, BN, BP, BR, BS, BT, BU, BV, BW, BX, BY, BZ, CA, CB, CC |  | 99.84 | A, B, C, H, I, K, L, M, N, P, R, S, T, U, W, X, Z, AA, AB, AC, AD, AE, AF, AH, AI, AJ, AK, AL, AM, AN, AO, AP, AR, AS, AT, AU, AV, AW, AX, AY, AZ, BA, BB, BF, BI, BJ, BK, BL, BP, BT, BU, BV, BW, BX, BY |
| **BR** | >1:12 |  | 97.94 | A, B, D, E, F, G, I, J, L, M, N, O, P, Q, R, S, T, V, W, X, Y, Z, AA, AB, AC, AD, AE, AF, AG, AH, AI, AK, AL, AM, AN, AO, AP, AQ, AR, AS, AT, AU, AV, AX, AY, BA, BB, BD, BE, BG, BH, BI, BJ, BK, BN, BO, BP, BQ,BV, BW, BX, BY, CA |  | 99.53 | A, B, E, F, G, I, K, L, M, N, P, Q, R, S, T, V, W, X, Z, AA, AB, AC, AD, AE, AF, AH, AI, AK, AL, AM, AN, AO, AP, AR, AS, AT, AU, AV, AW, AX, AY, AZ, BA, BB, BC, BF, BG, BJ, BK, BL, BP, BS, BT, BU, BV, BW, BX, BY, BZ, CA, CB, CC |
| **BS** | >1:12 |  | 97.23 | A, B, D, E, J, K, L, M, N, O, P, Q, S, T, W, X, Z, AA, AB, AC, AD, AE, AF, AG, AH, AI, AJ, AK, AL, AM, AN, AO, AP, AQ, AR, AS, AT, AU, AV, AX, AZ, BA, BB, BC, BG, BI, BK, BM, BN, BO, BP, BQ, BV, BW, BX, BY |  | 100.00 | A, B, C, H, I, K, L, M, N, P, R, S, T, U, W, X, Y, Z, AA, AB, AC, AD, AE, AF, AH, AI, AJ, AK, AL, AM, AN, AO, AP, AQ, AR, AS, AT, AU, AV, AW, AX, AY, AZ, BA, BB, BE, BF, BI, BJ, BK, BL, BM, BO, BP, BR, BT, BU, BV, BW, BX, BY |
| **BT** | >1:12 |  | 96.89 | A, B, E, G, I, J, L, N, O, P, Q, R, S, T, V, W, Z, AA, AB, AD, AE, AF, AG, AH, AI, AK, AL, AM, AN, AO, AP, AS, AT, AV, AX, AY, BA, BB, BD, BE, BH, BI, BJ, BN, BO, BP, BQ, BV, BW, BX, BY |  | 96.77 | A, B, D, E, F, G, J, K, L, O, Q, S, T, V, W, AA, AD, AE, AF, AG, AH, AI, AJ, AK, AL, AM, AO, AP, AQ, AS, AT, AV, AW, AX, BA, BB, BC, BD, BE, BG, BH, BI, BK, BL, BM, BN, BQ, BR, BS, BW, BX, BY, BZ, CA, CB, CC |
| **BU** | >1:12 |  | 98.59 | A, B, D, E, G, I, J, L, M, N, O, P, Q, R, S, T, V, W, X, Z, AA, AB, AC, AD, AE, AF, AG, AH, AI, AJ, AK, AL, AM, AN, AO, AP, AQ, AR, AS, AT, AU, AV, AX, AZ, BA, BB, BC, BD, BE, BG, BH, BI, BJ, BK, BN, BO, BP, BQ, BV, BW, BX, BY |  | 95.98 | B, D, E, F, G, J, L, M, Q, S, T, V, W, Z, AA, AD, AE, AF, AG, AH, AI, AJ, AK, AM, AP, AQ, AS, AT, AU, AV, AW, AX, AY, BA, BB, BC, BD, BE, BG, BH, BK, BL, BM, BQ, BR, BS, BV, BW, BX, BY, BZ, CA, CB, CC |
| **BV** | >1:12 |  | 93.08 | A, B, C, D, E, F, G, H, J, K, L, N, R, S, T, U, V, W, X, Y, Z, AA, AB, AE, AF, AG, AH, AI, AJ, AK, AL, AM, AN, AP, AR, AS, AT, AV, AW, AX, AY, AZ, BA, BB, BC, BD, BE, BF, BH, BJ, BL, BM, BN, BQ, BR, BS, BT, BU, BW, BX, BY, BZ, CA, CB, CC |  | 95.66 | A, B, D, E, F, G, H, I, J, K, L, N, O, Q, S, U, V, W, Y, AA, AB, AC, AD, AE, AF, AG, AH, AI, AJ, AK, AL, AO, AQ, AR, AS, AT, AV, AW, AX, BA, BB, BC, BD, BE, BG, BH, BI, BK, BL, BM, BN, BQ, BR, BS, BU, BW, BX, BY, BZ, CA, CB, CC |
| **BW** | <1:3 |  | 41.90 | A, C, D, E, F, G, H, I, J, K, L, M, N, O, P, Q, R, T, U, V, W, X, Y, Z, AA, AB, AC, AD, AE, AG, AH, AI, AJ, AK, AL, AM, AN, AO, AP, AQ, AR, AS, AT, AU, AV, AW, AX, AY, AZ, BA, BC, BD, BE, BF, BG, BH, BI, BJ, BK, BL, BM, BN, BO, BP, BQ, BR, BS, BT, BU, BV, BY, BZ, CA, CB, CC |  | 66.82 | A, B, C, D, E, F, G, H, I, J, K, L, M, N, O, P, Q, R, S, T, U, V, X, Y, Z, AB, AC, AE, AG, AH, AI, AJ, AL, AM, AN, AO, AP, AQ, AR, AS, AT, AU, AV, AW, AX, AY, AZ, BB, BC, BD, BE, BF, BG, BH, BI, BJ, BK, BL, BM, BN, BO, BP, BQ, BR, BS, BT, BU, BV, BZ, CA, CB, CC |
| **BX** | <1:3 |  | 43.37 | A, C, D, E, F, G, H, I, J, K, L, M, N, O, P, Q, R, T, U, V, W, X, Y, Z, AA, AB, AC, AD, AE, AG, AH, AI, AJ, AK, AL, AM, AN, AO, AP, AQ, AR, AS, AT, AU, AW, AX, AY, AZ, BA, BC, BD, BE, BF, BG, BH, BI, BJ, BK, BL, BM, BN, BO, BP, BQ, BR, BS, BT, BU, BV, BY, BZ, CA, CB, CC |  | 62.11 | A, B, C, D, E, F, G, H, I, J, K, M, N, O, P, Q, R, S, T, U, V, X, Y, Z, AA, AB, AC, AE, AG, AH, AI, AJ, AL, AM, AN, AO, AP, AQ, AR, AS, AT, AU, AV, AW, AY, AZ, BB, BC, BD, BE, BF, BG, BH, BI, BJ, BK, BL, BM, BN, BO, BP, BQ, BR, BS, BT, BU, BV, BZ, CA, CB, CC |
| **BY** | <1:3 |  | 48.72 | A, B, C, D, E, F, G, H, I, J, K, L, M, N, O, P, Q, R, T, U, V, X, Y, Z, AA, AB, AC, AD, AE, AG, AH, AI, AJ, AK, AL, AM, AN, AO, AP, AQ, AR, AS, AU, AW, AX, AY, AZ, BA, BC, BD, BE, BF, BG, BH, BI, BJ, BK, BL, BM, BN, BO, BP, BQ, BR, BS, BT, BU, BV, BW, BX, BZ, CA, CB, CC |  | 58.48 | A, B, C, D, E, F, G, H, I, J, K, M, N, O, P, Q, R, S, T, U, V, X, Y, Z, AA, AB, AC, AE, AG, AH, AI, AJ, AL, AM, AN, AO, AP, AQ, AR, AS, AT, AU, AV, AW, AY, AZ, BB, BC, BD, BE, BF, BG, BH, BI, BJ, BK, BL, BM, BN, BO, BP, BQ, BR, BS, BT, BU, BV, BZ, CA, CB, CC |
| **BZ** | >1:12 |  | 98.68 | A, B, D, E, G, I, J, L, M, N, O, P, Q, R, S, T, V, W, X, Y, Z, AA, AB, AC, AD, AE, AF, AG, AH, AI, AJ, AK, AL, AM, AN, AO, AP, AQ, AR, AS, AT, AU, AV, AX, AY, AZ, BA, BB, BC, BD, BE, BG, BH, BI, BJ, BK, BM, BN, BO, BP, BQ, BV, BW, BX, BY |  | 100.00 | A, B, C, H, I, K, L, M, N, P, R, S, T, U, W, X, Y, Z, AA, AB, AC, AD, AE, AF, AH, AI, AJ, AK, AL, AM, AN, AO, AP, AQ, AR, AS, AT, AU, AV, AW, AX, AY, AZ, BA, BB, BE, BF, BI, BJ, BK, BL, BM, BO, BP, BR, BT, BU, BV, BW, BX, BY |
| **CA** | >1:12 |  | 99.57 | A, B, D, I, J, K, L, M, N, O, P, Q, S, T, U, W, X, Z, AA, AB, AC, AD, AE, AF, AG, AH, AI, AJ, AK, AL, AM, AN, AO, AP, AQ, AR, AS, AT, AU, AV, AW, AX, AZ, BA, BB, BC, BG, BI, BK, BM, BN, BO, BP, BQ, BR, BV, BW, BX, BY, CB |  | 99.66 | A, B, C, I, K, L, M, N, P, R, S, T, U, W, X, Z, AA, AB, AC, AD, AE, AF, AH, AI, AJ, AK, AL, AM, AN, AO, AP, AR, AS, AT, AU, AV, AW, AX, AY, AZ, BA, BB, BF, BI, BJ, BK, BL, BO, BP, BR, BT, BU, BV, BW, BX, BY |
| **CB** | >1:12 |  | 98.26 | A, B, D, E, G, I, J, L, M, N, O, P, Q, R, S, T, V, W, X, Y, Z, AA, AB, AC, AD, AE, AF, AG, AH, AI, AJ, AK, AL, AM, AN, AO, AP, AQ, AR, AS, AT, AU, AV, AX, AY, AZ, BA, BB, BC, BD, BE, BG, BH, BI, BJ, BK, BN, BO, BP, BQ, BV, BW, BX, BY, CA |  | 99.98 | A, B, C, H, I, K, L, M, N, P, R, S, T, U, W, X, Y, Z, AA, AB, AC, AD, AE, AF, AH, AI, AJ, AK, AL, AM, AN, AO, AP, AQ, AR, AS, AT, AU, AV, AW, AX, AY, AZ, BA, BB, BE, BF, BI, BJ, BK, BL, BM, BO, BP, BR, BT, BU, BV, BW, BX, BY |
| **CC** | >1:12 |  | 97.63 | A, B, E, G, I, J, L, M, N, P, Q, R, S, T, V, W, Z, AA, AB, AC, AD, AE, AF, AG, AH, AI, AK, AL, AM, AN, AO, AP, AQ, AS, AT, AV, AX, BA, BB, BD, BE, BG, BH, BI, BJ, BN, BO, BP, BQ, BV, BW, BX, BY |  | 100.00 | A, B, C, H, I, K, L, M, N, P, R, S, T, U, W, X, Y, Z, AA, AB, AC, AD, AE, AF, AH, AI, AJ, AK, AL, AM, AN, AO, AP, AQ, AR, AS, AT, AU, AV, AW, AX, AY, AZ, BA, BB, BE, BF, BI, BJ, BK, BL, BM, BO, BP, BR, BT, BU, BV, BW, BX, BY |
